# Implementation of growth curves in a generic physiologically based kinetic model with a case study of perfluorooctanesulfonic acid (PFOS) in beef cattle, sheep, and chicken

**DOI:** 10.1007/s00204-026-04421-z

**Published:** 2026-05-02

**Authors:** David Inauen, Leonie Sophie Lautz, Aalbert Jan Hendriks, Ronette Gehring

**Affiliations:** 1 Institute for Risk Assessment Sciences, One Health Pharmacology, Faculty for Veterinary Medicine, Utrecht University, Yalelaan 104-106, 3584 CM Utrecht, The Netherlands; 2 ESQlabs GmbH, Am Sportplatz 7, 26683 Saterland, Germany; 3 Department of Environmental Science, Radboud University Nijmegen, Heyendaalseweg 135, 6525 AJ Nijmegen, the Netherlands

**Keywords:** PFOS, Livestock, Growth curve, Physiologically based kinetic model, Risk assessment

## Abstract

Perfluorooctanesulfonic acid (PFOS) is an environmental contaminant with a long half-life in animals. Risk assessment of PFOS in livestock and food tissue can be conducted with physiologically-based kinetic (PBK) models. For fast-growing livestock, changes in body weight affect the kinetics of PFOS primarily through tissue dilution, which needs to be considered when using a PBK model. A generic PBK model was used to predict tissue concentrations of PFOS in cattle, sheep, and chickens. The primary excretion routes were feces and urine, which were affected by enterohepatic circulation and saturable tubular reabsorption, respectively. Animal weight gain was modeled using the sigmoidal Richards curve. Model performance was validated by comparing simulations, with or without growth, to independent literature data. Blood and plasma concentrations of independent cattle and chicken data were accurately predicted. Liver and kidney concentrations were overpredicted by up to five-fold and three-fold, respectively, in cattle, and twelve-fold for liver in chickens. Liver concentrations in sheep were accurately predicted. Muscle concentrations were overpredicted in cattle by up to three-fold. Predictions using constant body weights differed from the growth PBK predictions by up to 80%, 12%, and 60% in cattle, sheep, and chicken tissue, respectively. Integration of growth in the PBK model led to improved accuracy in concentration predictions. Tissue concentration predictions could be improved using reliable partition coefficient estimates. The presented model closed gaps in previously developed models by integrating growth and PFOS-specific processes such as enterohepatic circulation and tubular reabsorption.

**Supplementary Information:**

The online version contains supplementary material available at 10.1007/s00204-026-04421-z.

## Introduction

A member of the per- and polyfluoroalkyl substances (PFAS) group, perfluorooctanesulfonic acid (PFOS) is a fluorinated organic compound having a sulfonated functional group and carbon backbones and is widely used in industrial and consumer applications (OECD 2013). Farms raising livestock can be contaminated by nearby factories or other installations, leading to potential impacts on human and animal health (Sunderland et al. 2019). Due to its high accumulation potential, PFOS is detected in many different samples, such as human tissues, soil and water samples, as well as in livestock and livestock products (Drew et al. 2021; Wee and Aris 2023; Mikolajczyk et al. 2024; Lupton et al. 2025). For livestock, limited studies on PFOS are available. A recent review on per- and polyfluoroalkyl substances in livestock and game species by Death et al. (2021) reported that half-lives of PFOS in different species could be up to years, and that PFOS concentrations were highest in blood, liver, and kidney, with minor accumulation in adipose tissue and muscle. Given the potential for PFOS contamination in feed, there is a need to translate external exposure to internal concentrations in livestock to enable robust human food safety assessment and to set evidence-based thresholds for environmental monitoring and regulation.

Growth modifies physiological determinants of PFOS kinetics, for example, food intake, tissue volumes, and blood flows, resulting in age-dependent concentrations. Growth dilution effects on PFOS concentrations through increased tissue volumes have previously been modeled in children (Koponen et al. 2018) or food chains (de Vos et al. 2008). In particular, growth dilution may affect the kinetics of fast-growing livestock such as cattle, sheep, and chickens. Kinetic models with static body weight (BW) do not describe growth dilution or other processes affected by growth, such as changing kidney performance. Neither do they take into account changes in exposure caused by growth. The effects of growth become especially apparent in slowly eliminated chemicals like PFOS. Elimination of PFOS is likely affected by enterohepatic circulation (EHC) and possibly saturable tubular reabsorption from urine, with feces being the main route of elimination (Kowalczyk et al. 2012; Lupton et al. 2014, 2015). Strong binding to plasma proteins may also explain the prolonged presence of PFOS in blood (Jones et al. 2003).

Physiologically based kinetic (PBK) models are typically used to calculate the transfer of chemicals from animal feed to food products, as they predict internal concentrations in various tissues based on external exposure. However, few such models are available for PFOS in livestock. A review by East et al. (2023) identified several empirical compartmental and PBK models for PFOS up to the year 2019, but only 3 out of 60 of those were in livestock. More recently, a PBK model for PFAS, including PFOS, was developed in adult beef/dairy cattle, using five compartments (Chou et al. 2023). Fischer et al. (2025) studied absorption and elimination kinetics in mice using a PBK model based on in vitro data. Furthermore, a PBK model for PFOS in humans of different life stages (Deepika et al. 2021), and a one-compartment model estimating tissue concentrations in cattle depending on variable food intake (Mikkonen et al. 2023) were published, both accounting for the growth of humans/animals.

Genericness of a model is important for food risk assessment due to the broad range of species and chemicals involved. While generic models with static BW exist (Chou et al. 2022; Dorne et al. 2023), previously developed PBK models that involve growth were non-generic and specific to a particular species and chemical. A generic PBK model in livestock, which incorporates growth, has yet to be implemented. For this study, the objective was to generalize PBK modeling by incorporating growth and simulate PFOS concentrations using the previously described physiological processes of recycling and reabsorption. To that end, a previously developed generic PBK model for livestock was modified (Lautz et al. 2020a, b; EFSA 2024). Sigmoidal growth functions for livestock were implemented to account for BW gain during the growth phase (Inauen et al. 2025), thus allowing simulation of changing exposure and dilution in tissue. Furthermore, EHC, saturable tubular reabsorption, and protein binding were implemented in a generic, not chemical-specific manner. The model was validated with case studies of PFOS in beef cattle, sheep, and chicken, where outcomes were compared to available in vivo data from the literature.

## Methods

### Model development

#### Model structure

**Fig. 1:**
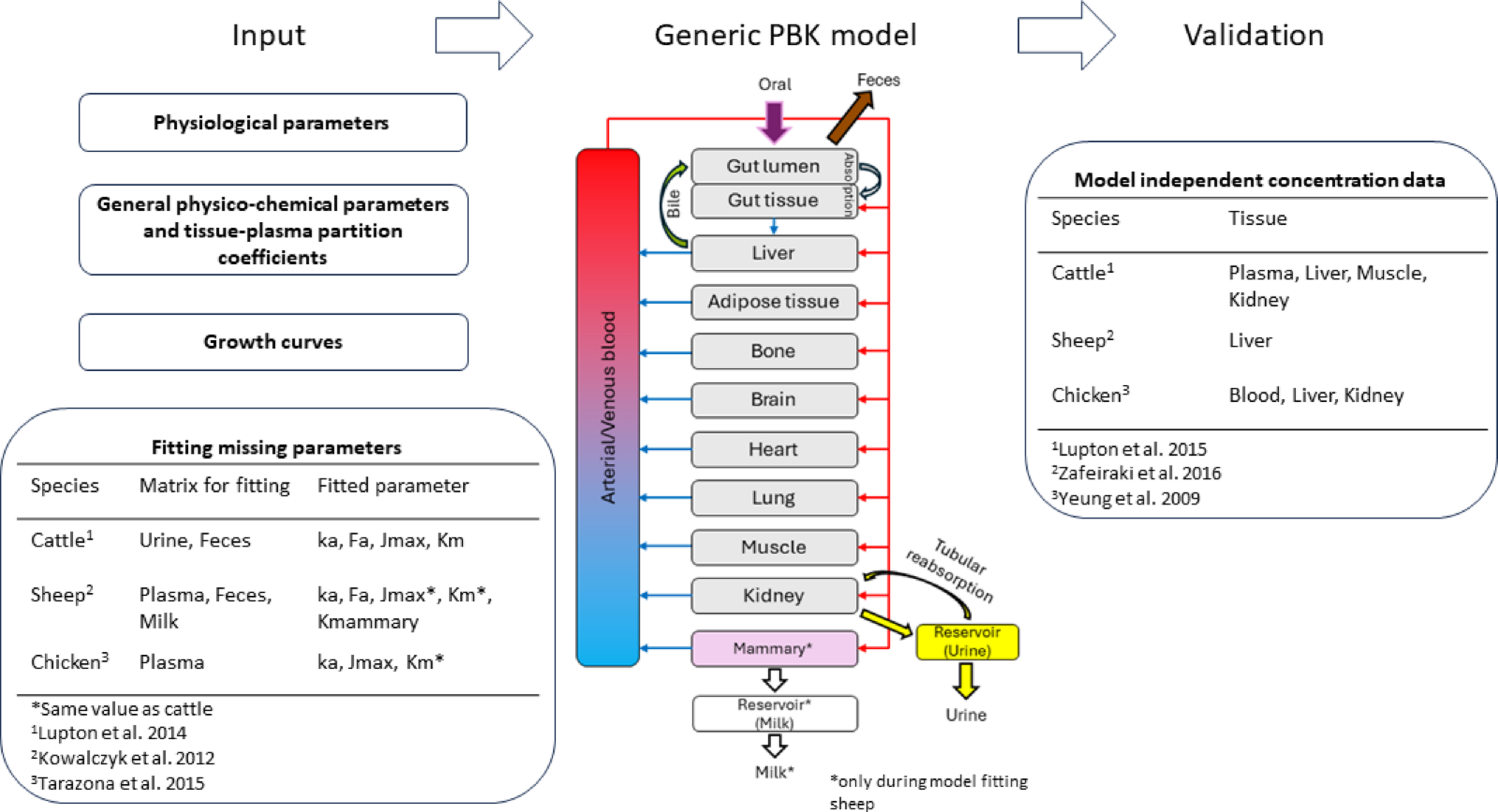
Model development and generic PBK structure for livestock

The model was developed following the guidelines for PBK modeling in risk assessment as set out by the World Health Organization (WHO 2010). The structure of the PBK model for PFOS was based on previously published generic models (Lautz et al. 2020a, b; EFSA 2024). The model structure was the same for all animal species, except for the addition of a mammary compartment during parameter fitting on sheep data. Species-specific differences were mediated through differing values of input parameters instead. The model consisted of a blood compartment, ten tissue compartments (gastrointestinal tract (GIT), liver, adipose tissue, bone, brain, heart, lung, muscle, kidney, and, for sheep, mammary), and two reservoir compartments for urine and milk (Fig. 1). The primary exposure route modeled was oral. Some modifications to the base models were made: The GIT consisted of two sub-compartments, gut lumen and gut tissue, enabling modeling of EHC as a separate process, which is an important driver of PFOS kinetics. Stomach was not included since it was not part of EHC, and absorption delay was not of primary concern in the study. A fraction (F_a_) of ingested PFOS was absorbed from gut lumen into gut tissue through a first-order process with an absorption rate constant (k_a_); the remainder 1 − F_a_ was excreted through feces. The enterohepatic cycle was then closed through constant biliary excretion of PFOS from the liver to gut lumen. Intestinal absorption and fecal excretion are physiologically dependent on intestinal transit time, but this was omitted in this model due to genericness considerations. Further, excretion of PFOS from the urine reservoir was assumed, representing urination. Thus, renally excreted PFOS did not continuously accumulate in the urine reservoir, which allowed modeling of PFOS reabsorption from the urine reservoir back into the kidneys. Excretion of PFOS from the milk reservoir compartment was assumed, representing milk secretion. All tissues were modeled as homogenous compartments with a blood flow-limited distribution. Distribution was modeled throughout the body via systemic circulation (Lautz et al. 2020a, b). PFOS was assumed to be eliminated via feces, urine, and, in the case of dairy sheep, milk. Metabolism was not considered a relevant route of elimination for PFOS and thus not included (European Food Safety Authority (EFSA) 2008). The mathematical representation of the PBK model is provided in the Online Resource (OR) Table S1. Computer implementation of all differential equations was done using the rxode2 package (v4.1.0) in the R software (v4.4.2) (R Core Team 2023; Fidler et al. 2024).

#### Physiological parameters

Physiological model parameters such as adult BW, tissue volumes (V_tissue_), cardiac output (CO), and tissue blood flows (Q_tissue_) for beef cattle, chicken, and sheep were taken from Dorne et al. (2023). Tissue volumes and blood flows were given as standardized fractions of BW and CO, respectively. Values for the glomerular filtration rate (CL_renal_), urine production (Q_urine_), milk production (Q_milk_), and bile formation (Q_bile_) were taken from literature, and all values and their references are summarized in OR Table S2.

#### General physico-chemical parameters and partition coefficients

Cattle: The blood–plasma ratio (BP) of 0.45 was assumed to be similar to that in humans (Ehresman et al. 2007). Fraction unbound in plasma (f_up_) was calculated as 0.006 based on a method for human plasma protein binding developed by Lobell and Sivarajah (2003), which agreed with Jones et al. (2003) who found that almost all PFOS in bovine serum was bound to albumins. Plasma partition coefficients (K_tissue_) for liver and muscle tissue were obtained as pooled means of tissue-to-blood/serum/plasma ratios using the inverse variance method for pooling (Vestergren et al. 2013; Drew et al. 2022; Lupton et al. 2025), see OR Table S3. K_kidney_ was obtained from Drew et al. (2022) and Lupton et al. (2025). Plasma partition coefficients of the remaining tissue were obtained by first calculating in silico partition coefficients for unbound PFOS in human plasma (K_tissue,u_), using the Rodgers and Rowland method (Rodgers and Rowland 2006), with the subsequent conversion of K_tissue_ = K_tissue,u_*fup. A pKa value smaller than one (Cheng et al. 2009) and a logP value of 5 (Kim et al. 2025) were assumed. Both f_up_ and the partition coefficients were calculated with the implementation provided in QIVIVE Tools (Punt et al. 2021). The physico-chemical parameters of the PBK model are provided in Table 1.

**Table 1:** Physico-chemical and growth PBK model parameters for cattle, sheep, and chicken

Type	Parameter	Unit	Beef cattle	Refs.	Sheep	Refs.	Chicken	Refs.
Physico-chemical	F_a_	–	0.954	Fitted	0.974	Fitted	0.990	Kowalczyk et al. (2020)
	k_a_	1/h	0.012	Fitted	0.098	Fitted	0.005	Fitted
	BP	–	0.45	Ehresman et al. (2007)	0.45	Ehresman et al. (2007)	0.45	Ehresman et al. (2007)
	f_up_	–	0.006	Lobell and Sivarajah (2003)	0.006	Lobell and Sivarajah (2003)	0.006	Lobell and Sivarajah (2003)
	CL_renal_	L/h/kg	0.13	Murayama et al. (2014)	0.108	Nesje et al. (1997)	0.15	Gasthuys et al. (2019)
	Jmax_invivo_	mg/h	4163	Fitted	4163	Cattle fitted value	64	Fitted
	Km	mg/L	24.27	Fitted	24.27	Cattle fitted value	24.27	Cattle fitted value
	K_liver_	–	1.25	Vestergren et al. (2013), Drew et al. (2022) and Lupton et al. (2025)	1.25	Cattle value	4.256	Yoo et al. (2009)
	K_kidney_	–	0.37	Drew et al. (2022) and Lupton et al. (2025)	0.37	Cattle value	0.325	Yoo et al. (2009)
	K_muscle_	–	0.06	Vestergren et al. (2013), Drew et al. (2022) and Lupton et al. (2025)	0.06	Cattle value	0.07	Rodgers and Rowland (2006)
	K_adipose_	–	0.05	Rodgers and Rowland (2006)	0.05	Rodgers and Rowland (2006)	0.05	
	K_bone_	–	0.1		0.1		0.1	
	K_brain_	–	0.05		0.05		0.0468	Yoo et al. (2009)
	K_heart_	–	0.16		0.16		0.16	Rodgers and Rowland (2006)
	K_gut_	–	0.16		0.16		0.16	
	K_lung_	–	0.21		0.21		0.21	
	K_mammary_	–	–	–	0.0031	Fitted (female dairy sheep)	–	–
Growth	A	kg	714	Inauen et al. (2025)	88	Inauen et al. (2025)	5.06 (Broiler) 1.91 (Laying chicken)	Inauen et al. (2025)
	W_0_	kg	38		4		0.04 (Broiler) 0.03 (Laying chicken)	
	kU	1/day	0.0014		0.003		0.0207 (Broiler) 0.0075 (Laying chicken)	
	d	–	0.67		0		Gompertz (Broiler) 0.75 (Laying chicken)	

Sheep: BP and f_up_ for sheep were assumed to be the same as for beef cattle. Likewise, plasma K_tissue_ values for liver, kidney, and muscle were assumed to be the same as those for beef cattle due to their similarity in tissue composition (Lautz et al. 2024). The K_tissue_ values for other sheep tissues were calculated with QIVIVE Tools as described for cattle.

Chicken: BP and f_up_ were assumed to be the same as in beef cattle. K_tissue_ values for liver, kidney, and brain were obtained from Yoo et al. (2009) by dividing tissue concentrations by blood concentrations given at the end of a 28-day subcutaneous exposure phase, see also OR Table S4. Tissue-blood ratios were converted to plasma K_tissue_ values by multiplication with BP. Other K_tissue_ values were calculated with QIVIVE Tools as previously described. F_a_ was assumed to be 0.99 as Kowalczyk et al. (2020) found that in laying hens 99% of absorbed PFOS was transferred into eggs.

### Model refinement

#### Growth model

BW gain was modeled by an application of the Richards family (Richards 1959) of sigmoidal growth curves. The Richards growth curve (Eq. 1), dependent on time t, was used in the unified form as proposed by Tjørve and Tjørve (2017):

1 $$\begin{aligned} {\mathrm{Growth}}\left( {\mathrm{t}} \right) & = {\mathrm{A}}*\left( {1 + \left( {\left( {{\mathrm{W}}_{0} /{\mathrm{A}}} \right)^{{\left( {1 - {\mathrm{d}}} \right)}} - 1} \right)} \right. \\ & \quad \left. {*\exp \left( { - {\mathrm{kU}}*{\mathrm{t}}/{\mathrm{d}}^{{{\mathrm{d}}/\left( {1 - {\mathrm{d}}} \right)}} } \right)} \right)^{{1/\left( {1 - {\mathrm{d}}} \right)}} \\ \end{aligned}$$

The curve parameters A (kg) and W0 (kg) denote the asymptotic (adult) weight and birth weight, respectively, while kU (1/day) and d (unitless) denote the slope at the inflection point and a shape parameter, respectively. For the special case when d approaches one, known as the Gompertz curve (Tjørve and Tjørve 2017), the equation becomes (Eq. 2):

2 $${\mathrm{Growth}}\left( {\mathrm{t}} \right) = {\mathrm{A}}*\left( {{\mathrm{W}}_{0} /{\mathrm{A}}} \right)^{{\exp \left( { - {\mathrm{exp}}\left( 1 \right)*{\mathrm{kU}}*{\mathrm{t}}} \right)}}$$

The growth parameters were assumed not to be breed-specific but generic (Table 1). For beef cattle, sheep, and laying chickens, the unified Richards curve was used, while for broilers, the Gompertz curve was used (Inauen et al. 2025).

The age (t_0_) of animals at the beginning of a study was found by obtaining the root of the function $\mathrm{f}\left(\mathrm{t}\right)=\mathrm{Growth}\left(\mathrm{t}\right)-{\mathrm{BW}}_{\mathrm{study}}$, using the BW at the start of a study (BW_study_). The solution was unique because of the strict monotonicity of the Richards curve. Root finding was implemented with the ‘bisect’ function of the R package pracma (v2.4.4) (Borchers 2023). During simulation, BW at time t of a study was given by Eq. 3:

3 $${\mathrm{BW}}\left(\mathrm{t}\right)={\mathrm{Growth}}\left({\mathrm{t}}_{0}+\mathrm{t}\right)$$

CO was scaled by a fixed factor of BW, and tissue volumes and blood flows were scaled at fixed proportions to BW and CO, respectively. Further, renal clearance and bile flow were scaled with BW. Predicted BW values can be found in the OR Table S5 and Fig. S1.

#### Parameter fitting

The PBK model was refined by fitting parameters to literature data in beef cattle, chicken, and sheep, using studies with mainly short-term observation data (Table 2). Parameter fitting for cattle was conducted in two steps: First, k_a_, F_a_, and a linear rate constant (k_reabsorb_) for renal reabsorption (Eqs. 4–5) were fitted to fecal and urine PFOS accumulation data from Lupton et al. (2014).

**Table 2:** Literature data for fitting or validation of the model

Data	Species	n	Study duration (days)	BW mean at study begin (kg)	Exposure	Exposure duration (days)	Fitted parameters	Matrix for fitting	Predicted matrix	Refs
Fitting	Male beef cattle	3	28	329	8000 μg/kg	Single bolus	k_a_, F_a_, Jmax_invivo_, Km	Feces, urine	Plasma, liver, kidney, muscle	Lupton et al. (2014)
	Female dairy sheep	2	42	63	1.16 μg/kg/day 1.45 μg/kg/day	21	k_a_, F_a_, K_mammary_, Jmax_invivo_^a^, km^a^	Plasma, feces, milk	Liver, kidney, muscle	Kowalczyk et al. (2012)
	Male broiler chicken	6	231	1.5	0.085 μg/kg/day	102	k_a_, Jmax_invivo_, km^a^	Plasma^b^	–	Tarazona et al. (2015)
Validation	Male, female beef cattle	2–4	343	308	98 μg/kg 9090 μg/kg	Single bolus		–	Plasma, liver, kidney, muscle	Lupton et al. (2015)
	Non-dairy sheep	8	112	24	0.0208 μg/kg/day	56 or 112		–	Liver	Zafeiraki et al. (2016)
	Male laying chicken	12	42	0.04	100 or 1000 μg/kg/dose	21 (ten doses)		–	Blood, liver, kidney	Yeung et al. (2009)

^a^Same as cattle

^b^Originally serum

4 $$\begin{aligned} \frac{{{\mathrm{dA}}_{{{\mathrm{kidney}}}} }}{{{\mathrm{dt}}}} & = {\mathrm{Q}}_{{{\mathrm{kidney}}}} *\left( {{\mathrm{C}}_{{{\mathrm{art}}}} - {\mathrm{CV}}_{{{\mathrm{kidney}}}} } \right) - {\mathrm{CL}}_{{{\mathrm{renal}}}} *{\mathrm{BW}} \\ & \quad *\frac{{{\mathrm{CV}}}}{{{\mathrm{BP}}}}*{\mathrm{f}}_{{{\mathrm{up}}}} + {\text{Rate of renal reabsorption}} \\ \end{aligned}$$

5 $$\begin{aligned} & {\text{Linear rate of renal reabsorption}} \\ & \quad = {\mathrm{k}}_{{{\mathrm{reabsorb}}}} *{\mathrm{C}}_{{{\mathrm{urine}}}} \\ \end{aligned}$$

A_kidney_ denoted the amount of PFOS in kidney, C_art_ and CV_kidney_ the concentration of inflowing, respectively outflowing blood, CV the concentration in venous blood, and C_urine_ the concentration in the urine reservoir. The parameters were not fitted to observed plasma concentrations of Lupton et al. (2014) due to high variation in that data; however, the plasma data were used to ensure that the absorption phase and maximal concentrations were matched by the fitted model. Fitting was conducted using a grid of initial parameter values. The fit with the smallest relative standard errors in the parameter estimates was considered optimal. As the next step, the estimates of k_a_ and F_a_ were fixed, the linear reabsorption was replaced by saturable reabsorption implemented via the Michaelis–Menten equation (Eq. 6), and the maximal rate of transport (Jmax_invivo_) and the Michaelis constant for transport (Km) were fitted to fecal and urine data.

6 $$\begin{aligned} & {\text{Saturable rate of renal reabsorption}} = \frac{{{\mathrm{Jmax}}_{{{\mathrm{invivo}}}} }}{{{\mathrm{Km}} + {\mathrm{C}}_{{{\mathrm{urine}}}} }} \\ & \quad *{\mathrm{C}}_{{{\mathrm{urine}}}} *\left( {\frac{{{\mathrm{BW}}_{{{\mathrm{species}}}} \left( {\mathrm{t}} \right)}}{{70}}} \right)^{{0.75}} \\ \end{aligned}$$

Initial values for the fitting of Jmax_invivo_ and Km were taken from human organic anion transporter 4 (OAT4)-mediated uptake reported by Louisse et al. (2023) (see OR 1). Since the initial Jmax_invivo_ value was from humans, the rate was allometrically scaled to the BW of the species in question and consequently, the saturable reabsorption was dependent on growth. For dairy sheep, k_a_, F_a_ and K_mammary_ were fitted to PFOS plasma concentration data and fecal and milk accumulation data from Kowalczyk et al. (2012); the data consisted of two individual sheep with different exposures (1.16 and 1.45 μg/kg/day). Because urinary excreted amounts of PFOS were not quantifiable, the fitted Jmax_invivo_ value obtained from cattle was used for sheep. For chicken, initially k_a_ was fitted to plasma concentration data found in Tarazona et al. (2015), assuming linear renal reabsorption. Subsequently, the estimated k_a_ was fixed, and Jmax_invivo_ was fitted using a grid of initial values. Fitting was conducted using the R package nlmixr2 (v3.0.2) with the ‘lbfgsb3c’ optimization algorithm (Fidler 2025). 

#### Parameter variability

Literature values of K_liver_, K_kidney_, and K_muscle_ in cattle varied, thus 95% confidence intervals were calculated from a random-effects model (OR 1 Table S3) and 100 values for each of the three parameters were sampled from a uniform distribution on those intervals. This method did not reflect a real population but presented the possible range of predicted values arising from different combinations of K_tissue_ values obtained from literature data. The same procedure was conducted with sheep. The range of predicted values was plotted along with the median value. For chicken simulations, no variability was introduced due to a lack of multiple literature references.

### Global sensitivity analysis

A global sensitivity analysis (GSA) was implemented using Sobol’ indices (Sobol’ 2001), which quantify the influence of a parameter on the variance of a kinetic endpoint, in isolation (first-order indices), or with interaction with other parameters (total indices). For each dataset used for fitting, indices of all physico-chemical parameters as listed in Table 1 were quantified with endpoints area under the curve (AUC) of the blood concentration, and the liver concentration (C_liver_), representative for tissues. Each parameter was uniformly sampled 100 times within ± 10% of the default value; the maximally possible value of F_a_ was set to one. Indices were computed using the extended Fourier amplitude sensitivity testing method implemented in the function ‘fast99’ of the R package ‘sensitivity’ (v1.30.1) (Saltelli et al. 1999; Iooss et al. 2023).

### Validation

Studies with long-term observation data independent of the model development were chosen for validation of model performance (Table 2). Those studies included growing animals to highlight the effect of growth dilution: Lupton et al. (2015) for beef cattle, Zafeiraki et al. (2016) for non-dairy sheep, and Yeung et al. (2009) for male layer-type chicken. No milk production was assumed in the non-dairy sheep, and tissue volume and blood flow fractions were rescaled without the mammaries. For sheep, additional predictions were made, which, instead of fitted values from sheep data, employed the fitted k_a_ and F_a_ values from the cattle data. To quantify the influence of growth, predictions with constant BW after the exposure phase (static model) were compared with the growth model.

For time-concentration curves, the geometric mean fold error (GMFE) was reported (Eq. 7):

7 $${\mathrm{GMFE}}={10}^{\frac{{\sum }_{\mathrm{i}=1}^{\mathrm{n}}\left|\mathrm{log}10\left(\frac{{y}_{i,pred}}{{y}_{i,obs}}\right)\right|}{n}}$$

with y_i,obs_ and y_i,pred_ the i-th mean observed and median predicted plasma/blood/tissue concentration values, respectively. As evaluation metric for concentrations at singular time points, fold errors (FE) were calculated as follows (Eq. 8):

8 $${\mathrm{FE}}_{\mathrm{i}}=\frac{{y}_{i,pred}}{{y}_{i,obs}}$$

with y_i,obs_ and y_i,pred_ the i-th mean observed and median predicted tissue concentration values, respectively. Also, the percentage of predicted data points between 0.5 and 2 FE (% two-fold) was given.

## Results

### Cattle

**Fig. 2:**
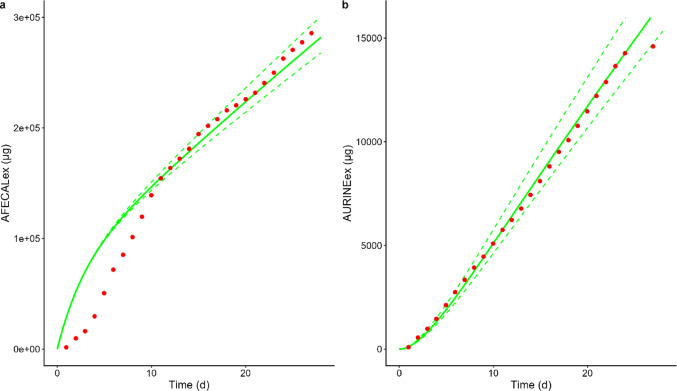
Model fit to accumulated excreted PFOS in feces (**a**) and urine (**b**) of beef cattle (Lupton et al. 2014). The red dots are the mean observed data points. The green curves represent the median simulation results including growth, and the dashed lines are the results of the introduced variation of the partition coefficients

In the first 10 days after dosing, the fitted model overestimated the amount of fecally excreted PFOS, but fitted the later time points well (Fig. 2a). The initial overestimation came from the fact that fecal excretion was implemented as first-order process. Urine data were fitted well, with overprediction of a data point on day 28 (Fig. 2b).

**Fig. 3:**
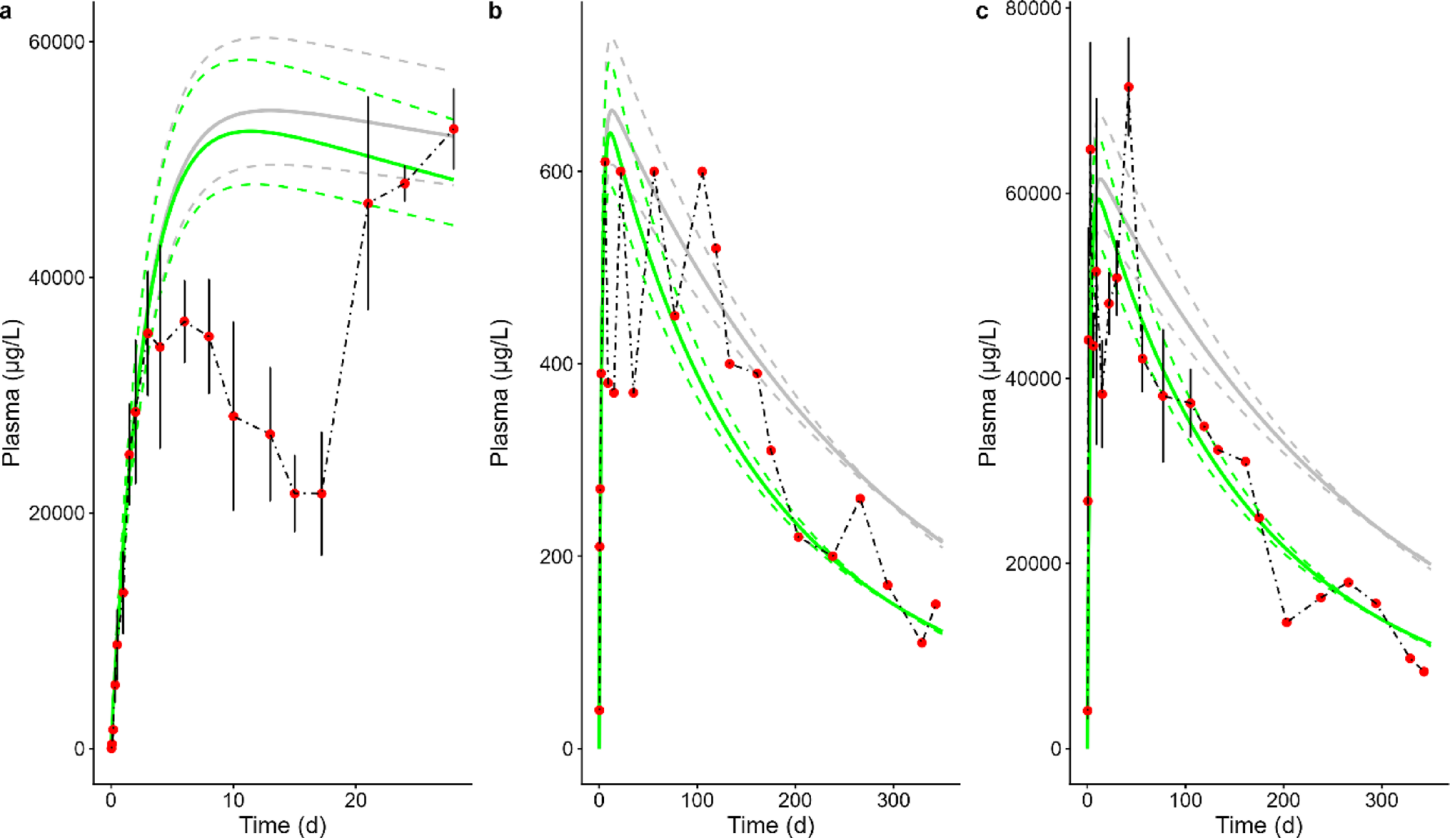
Observed plasma data and simulated time-concentration curves after single oral bolus exposure. Fitting data in beef cattle: **a** 8000 μg/kg (Lupton et al. 2014). Validation data in beef cattle: **b** 98 μg/kg, **c** 9090 μg/kg (Lupton et al. 2015). The red dots are the mean observed data points ± standard deviation, connected by dot–dashed lines. The green curves represent the median simulation results including growth and the gray curves are the results where BW was held constant after exposure. The dashed curves are the results of the introduced variation of the partition coefficients

**Fig. 4:**
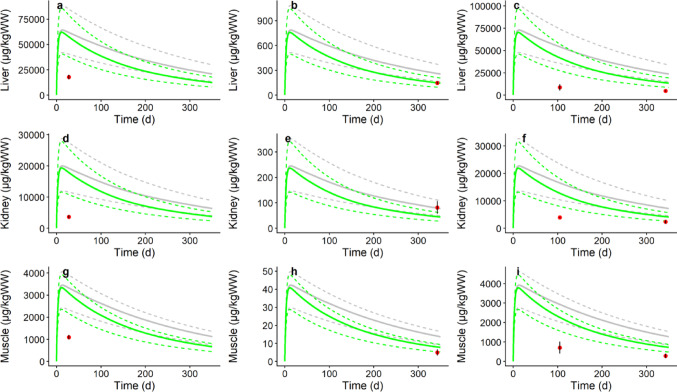
Observed tissue data and simulated time-concentration curves after single oral bolus exposure. Fitting data in beef cattle 8000 μg/kg: **a** liver, **d** kidney, **g** muscle (Lupton et al. 2014). Validation data in beef cattle 98 μg/kg: **b** liver, **e** kidney, **h** muscle, and 9090 μg/kg **c** liver, **f** kidney, **i** muscle (Lupton et al. 2015). Tissue concentrations are given per kg wet weight (WW). The red dots are the mean observed data points ± standard deviation. The green curves represent the median simulation results including growth and the gray curves are the results where BW was held constant after exposure. The dashed curves are the results of the introduced variation of the partition coefficients

Plasma concentrations in cattle were predicted accurately (Fig. 3), whereas tissue concentrations were generally overpredicted (Fig. 4). The plasma concentration GMFEs for the fitting and validation studies were 1.8 and 1.3, respectively, as presented in Tables 3 and 4. Predicted plasma concentrations reached up to the maximum observed concentrations for both the fitting (Fig. 3a) and validation data (Fig. 3b,c), and matched the elimination phase of the validation data (Lupton et al. 2014, 2015). When assuming constant BW, the GMFE in the validation data increased by up to 20%.

**Table 3:** Evaluation metrics of fitted/predicted concentrations in the fitting data

Species	Male beef cattle	Female dairy sheep	Female dairy sheep	Male broiler chicken
n animals	3	1	1	6
Exposure	8000 μg/kg	1.16 μg/kg/day	1.45 μg/kg/day	0.085 μg/kg/day
Plasma	1.78 (73.7%)	1.40 (87.5%)	1.50 (76.9%)	1.18 (92.0%)
Plasma, BW constant	1.80 (68.4%)	1.39 (87.5%)	1.50 (76.9%)	1.18 (92.0%)
Measurement time points (days)	28	42	21	–
Liver	3.21	0.21	0.22	–
Liver, BW constant	3.46	0.22	0.22	–
Kidney	4.83	0.34	0.28	–
Kidney, BW constant	5.20	0.36	0.28	–
Muscle	2.81	0.42	0.40	–
Muscle, BW constant	3.02	0.44	0.40	–
Refs	Lupton et al. (2014)	Kowalczyk et al. (2012)	Kowalczyk et al. (2012)	Tarazona et al. (2015)

For time-concentration curves, the GMFE is given with % two-fold in parentheses. For singular time point measurements, the time of measurement is given as well as the FE

**Table 4:** Evaluation metrics of predicted tissue concentrations in the validation data

Species	Male beef cattle	Female beef cattle	Sheep	Sheep	Male laying chicken	Male laying chicken
n animals	2	4	4	4	6	6
Exposure	98 μg/kg	9090 μg/kg	0.0208 μg/kg/day up to day 56	0.0208 μg/kg/day up to day 112	100 μg/kg/dose	1000 μg/kg/dose
Plasma or Blood^1^	1.27 (100%)	1.27 (91.3%)	–	–	1.66 (83.3%)	1.15 (100%)
Plasma or Blood^a^, BW constant	1.33 (95.5%)	1.51 (78.3%)	–	–	1.42 (83.3%)	1.50 (83.3%)
Measurement time points (days)	343	105, 343	56–112	0–112	21, 42	21, 42
Liver	1.00	4.78, 2.93	1.26 (100%)	1.31 (100%)	9.00, 11.08	7.61, 11.53
Liver, BW constant	1.76	6.19, 5.13	1.41 (100%)	1.31 (100%)	9.00, 22.44	7.61, 23.33
Kidney	0.55	3.23, 1.79	–	–	0.75, 1.08	0.76, 1.14
Kidney, BW constant	0.97	4.18, 3.13	–	–	0.75. 2.18	0.76, 2.31
Muscle	1.60	3.18, 2.31	–	–	–	–
Muscle, BW constant	2.81	4.11, 4.03	–	–	–	–
Refs	Lupton et al. (2015)	Lupton et al. (2015)	Zafeiraki et al. (2016)	Zafeiraki et al. (2016)	Yeung et al. (2009)	Yeung et al. (2009)

For time-concentration curves, the GMFE is given with % two-fold in parentheses. For singular time point measurements, the time of measurement is given as well as the FE. For sheep liver time-concentration, the time range is given. ^a^Male laying chicken

Tissue concentrations were overpredicted, especially in the liver, by up to a factor five. An exception was the underprediction of kidney concentration at 343 days of the 98 μg/kg study, with an FE of 0.6. Tissue observations at later time points were better predicted than earlier observations. The prediction intervals introduced by varying K_tissue_ values in the model did not capture the observed tissue data points. For predictions with static BW, differences in FEs increased with study duration: At 105 days of the 9090 μg/kg study, the liver FE increased by 30% compared to the growth model, and at 343 days, it had increased by 80%. FEs of other tissues scaled in similar proportions. As the exposures were single bolus, BW was fixed right at the start of the study for those predictions, thus the increases in tissue concentration corresponded to the proportional increase in BW. Note that the plasma data of Lupton et al. (2014) in Fig. 3a were not used for fitting, while the feces and urine data were.

### Sheep

**Fig. 5:**
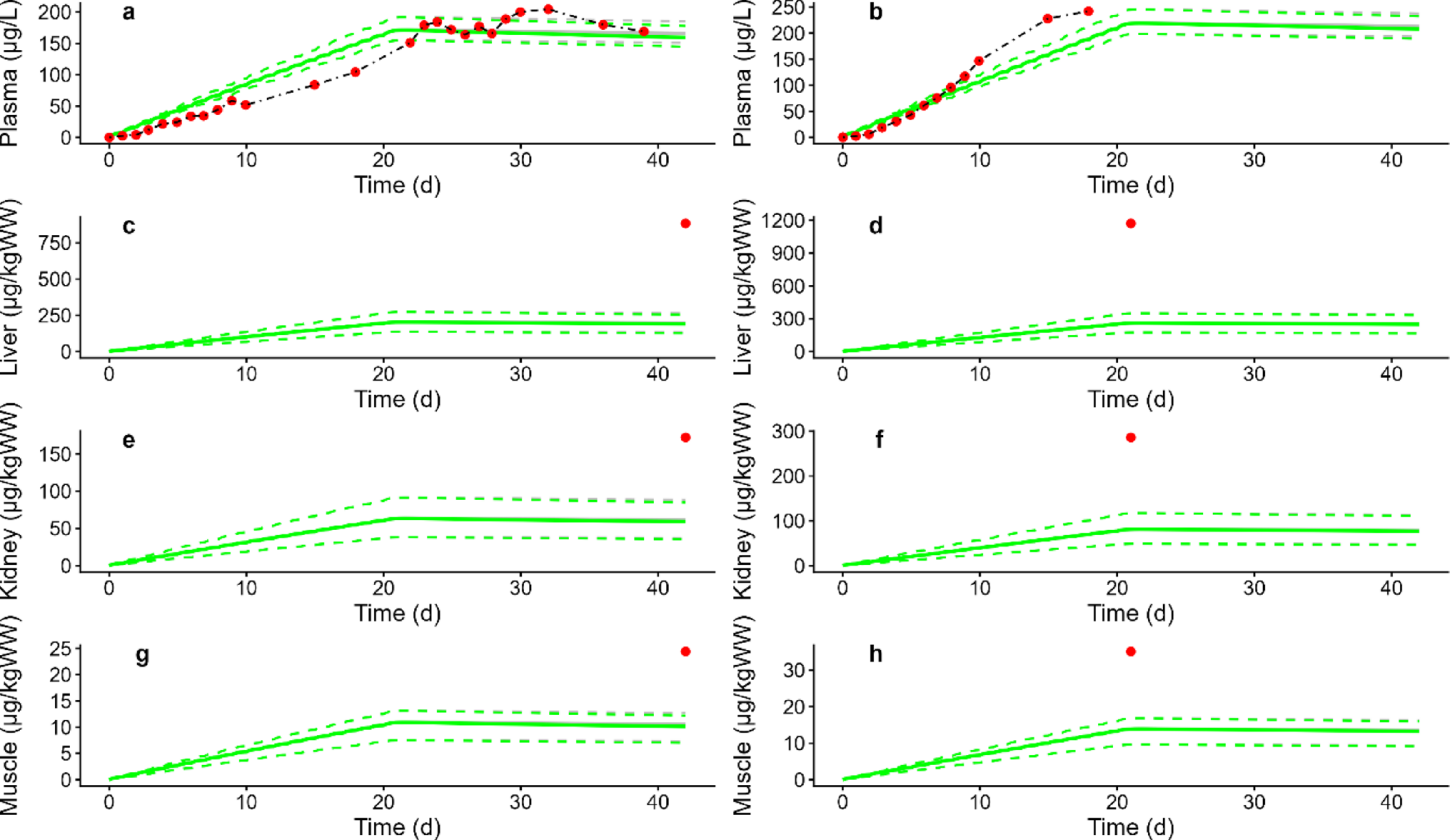
Observed plasma and tissue data, and simulated time-concentration curves after 21 days oral exposure. Fitting data in dairy sheep 1.16 μg/kg/day: **a** plasma, **c** liver, **e** kidney, **g** muscle, and 1.45 μg/kg/day: **b** plasma, **d** liver, **f** kidney, **h** muscle (Kowalczyk et al. 2012). Tissue concentrations are given per kg wet weight (WW). The red dots are the mean observed data points, connected by dot-dashed lines. The green curves represent the median simulation results including growth and the gray curves are the results where BW was held constant after exposure. The dashed curves are the results of the introduced variation of the partition coefficients

**Fig. 6:**
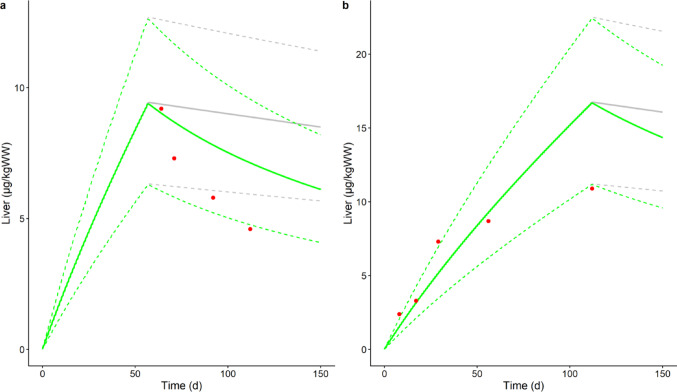
Observed liver data and simulated time-concentration curves after multiple oral exposures. Validation data in non-dairy sheep: 0.0208 μg/kg/day, **a** 56 days exposure, **b** 112 days exposure (Zafeiraki et al. 2016). Tissue concentrations are given per kg wet weight (WW). The red dots are the mean observed data points. The green curves represent the median simulation results including growth and the gray curves are the results where BW was held constant after exposure. The dashed curves are the results of the introduced variation of the partition coefficients

Tissue concentrations of the fitting data were underpredicted, while the liver concentrations of the validation data were accurately predicted. The plasma profiles of neither sheep in the fitting study were accurately fitted: Fig. 5a and b show that during exposure, the plasma profiles of the 1.16 μg/kg/day and 1.45 μg/kg/day sheep were, respectively, over- and underpredicted. However, maximum plasma concentrations were reached by the fitted curve. Liver, kidney, and muscle concentrations were underpredicted (Fig. 5c–h); liver was predicted least accurately, with a more than four-fold underprediction (Table 3). The predictions without growth after exposure performed slightly better; differences in BW were minimal as the animals had already reached the adult stage. The fits underpredicted PFOS excreted in feces and milk for both sheep (OR Table S6). In the validation data of Zafeiraki et al. (2016), predicted liver concentrations were in the range of the observed data with a GMFE around 1.3 for both the 56-day and 112-day exposure studies (Table 4). Figure 6a shows that the simulated elimination from the liver was slower than the observed elimination. When BW was held constant during depuration, the liver concentrations remained almost constant, and the liver GMFE increased by more than 10%.

Using F_a_ and k_a_ values fitted from cattle data of Lupton et al. (2014), the plasma concentration data of the 1.16 μg/kg/day sheep were well-predicted, while the data of the 1.45 μg/kg/day sheep were underpredicted with a GMFE of 1.53 (OR Fig. S4a,b, OR Table S7). In the validation study, the predicted liver concentrations (OR Fig. S5) were lower than the predictions from the model fitted to sheep data, but still in the range of the observed data.

### Chicken

**Fig. 7:**
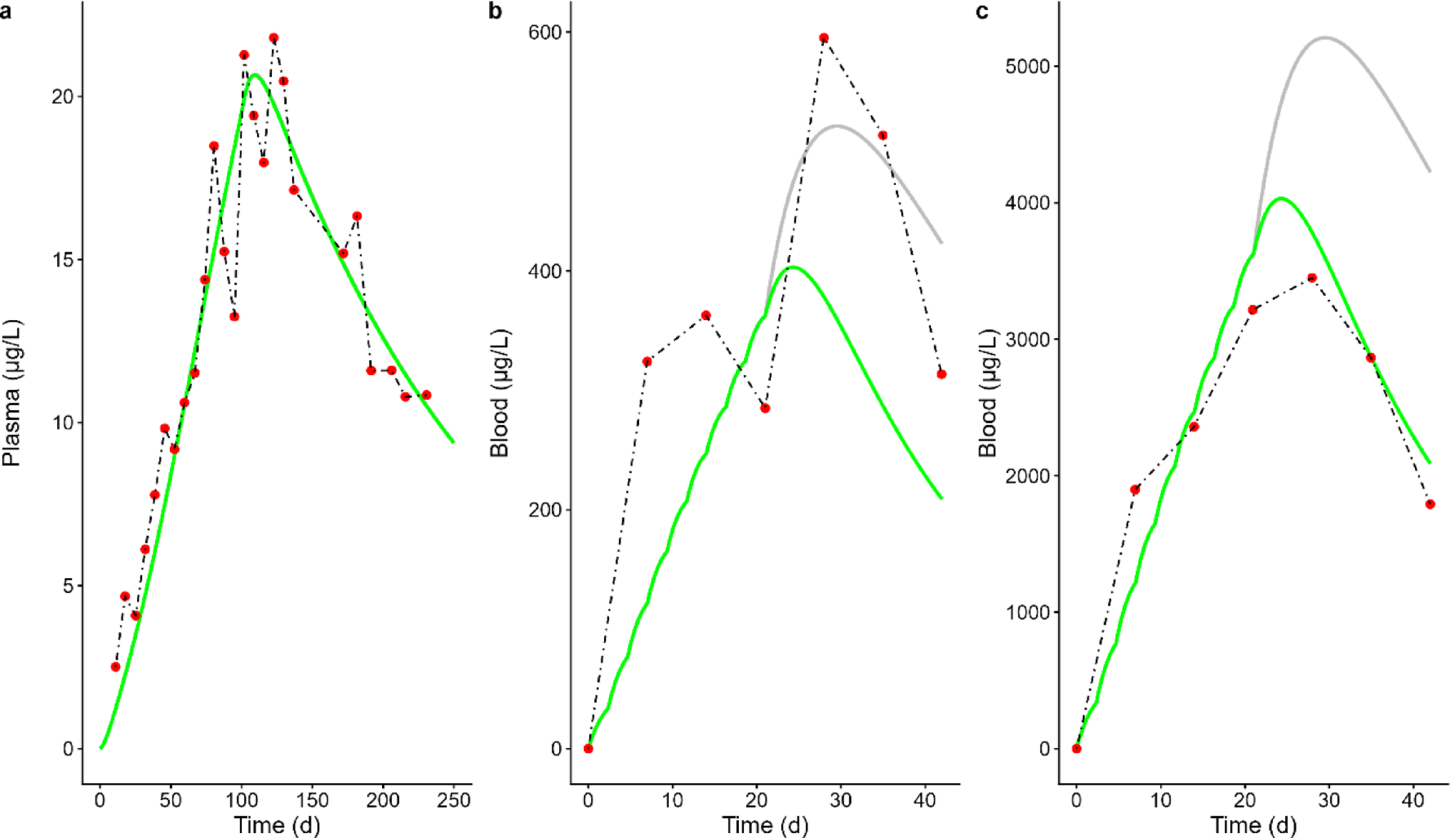
Observed plasma and blood data and simulated time-concentration curves after multiple oral exposures. Fitting data in broiler chicken: **a** 0.085 μg/kg/day (Tarazona et al. 2015). Validation data in male laying chicken: **b** 100 μg/kg/dose, **c** 1000 μg/kg/dose for a total of ten doses (Yeung et al. 2009). The red dots are the mean observed data points, connected by dot-dashed lines. The green curves represent the median simulation results including growth and the gray curves are the results where BW was held constant after exposure

**Fig. 8:**
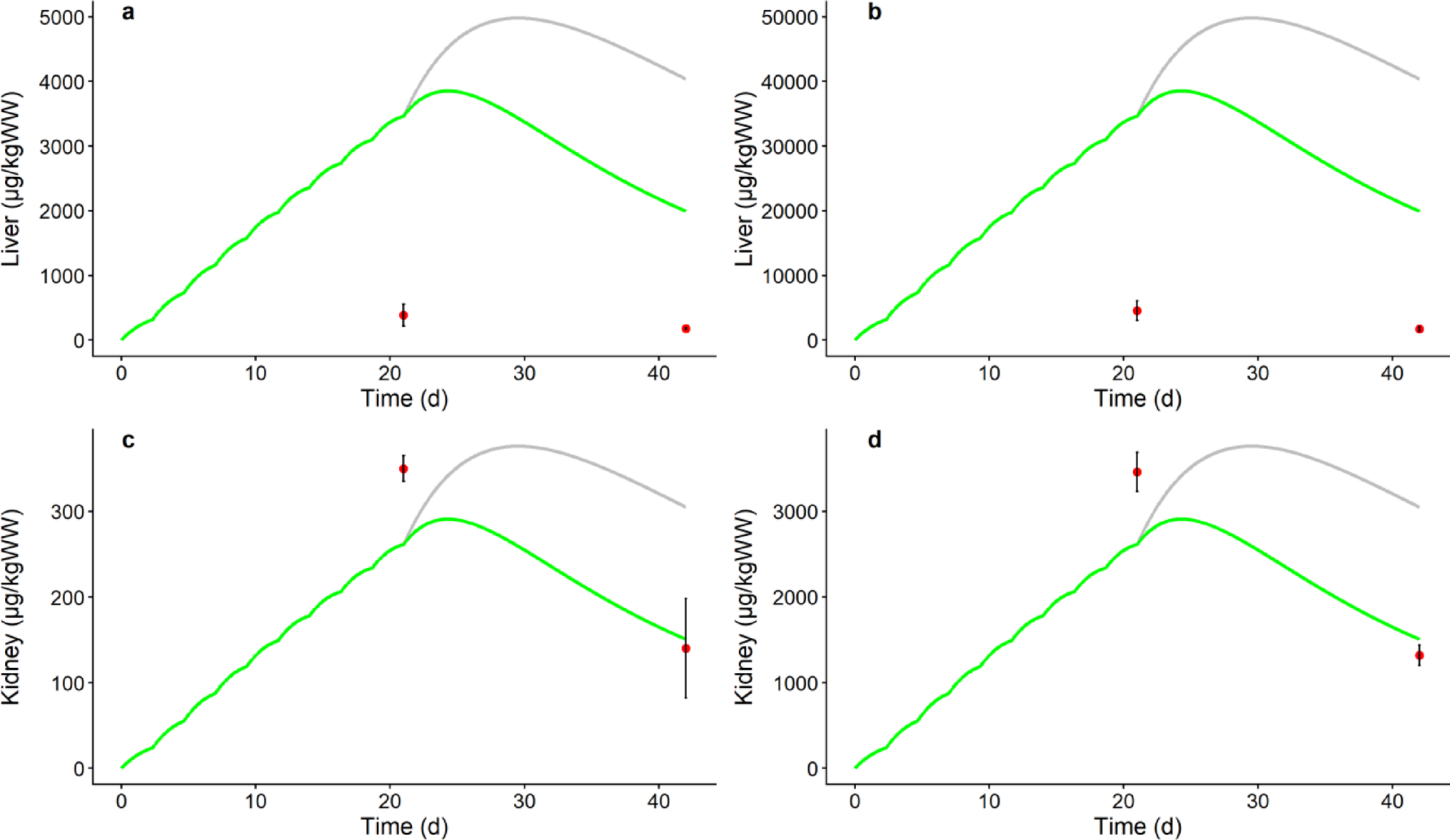
Observed tissue data and simulated time-concentration curves after multiple oral exposures. Validation data in male laying chicken 100 μg/kg/dose: **a** liver, **c** kidney, and 1000 μg/kg/dose: **b** liver, **d** kidney for a total of ten doses (Yeung et al. 2009). The red dots are the mean observed data points ± standard deviation. The green curves represent the median simulation results including growth and the gray curves are the results where BW was held constant after exposure

The fitted model agreed well with the plasma concentration of the fitting data (Tarazona et al. 2015), with a GMFE of 1.2 (Table 3). Predictions with static BW were identical since the animals had reached maturity at the end of the exposure phase (Fig. 7a). Overall, in the validation data, blood and kidney concentrations were accurately predicted, while liver concentrations were strongly overpredicted. The blood concentration prediction (Fig. 7b) of the 100 μg/kg dosing validation study (Yeung et al. 2009) was underpredicted with a GMFE of 1.7, while the 1000 μg/kg dosing validation study (Fig. 7c) was accurately predicted with a GMFE of 1.2 (Table 4). Liver concentrations for both dosing studies were overpredicted by up to a factor of 12. Kidney concentration predictions were in the two-fold error range of the observations (Fig. 8). When assuming static BW, the GMFE for the 100 μg/kg study was lower by 15% than the growth model GMFE, while for the 1000 μg/kg study, it was higher by 30%. Due to the high estimated absorption half-life of ln(2)/k_a_ ≈ 139 h, concentrations in the static BW model continued to increase after the end of the exposure phase.

### Global sensitivity analysis

GSA of the beef cattle, dairy sheep and broiler studies (Kowalczyk et al. 2012; Lupton et al. 2014; Tarazona et al. 2015) showed that F_a_, k_a_, and BP were the most influential parameters for AUC variance during the exposure phase; the influence of F_a_ on the variance of the AUC continuously increased over time while the influence of the latter two decreased (OR Figs. S6–S8). K_tissues_ were of marginal importance. First-order and total indices were similar, indicating that interaction effects were minimal. For the liver concentrations, F_a_, k_a_, and K_liver_ were the most influential parameters, with K_liver_ initially increasing and subsequently decreasing influence. In broilers, CL_renal_, f_up_, Jmax, and Km additionally contributed to the variance of both AUC and liver concentration.

## Discussion

Plasma concentrations of cattle, sheep, and broilers were well predicted, with GMFEs below 2. Simulation of growth increased the accuracy of predictions, with differences in tissue FEs of up to 80% over longer time spans. Predicted tissue concentrations were outside of the range of the observed data, with the largest errors found in liver tissue. GSA found that predicted tissue concentrations were mostly influenced by F_a_, k_a_, BP, and K_tissue_ values. The results in cattle suggest that excreted PFOS in feces and urine prove to sufficiently model the plasma absorption and elimination phases, but not the distribution phase.

### Cattle

Tissue concentrations were overpredicted by a factor of up to five. The main reason for inaccuracy was the choice of the model’s K_tissue_ values, calculated as tissue-plasma concentration ratios, which may deviate from the observed data of the validation studies. For example, K_liver_ values chosen for beef cattle stemmed from animals at steady-state concentrations (Vestergren et al. 2013; Drew et al. 2022; Lupton et al. 2025), with observed mean liver-plasma concentration ratios between 0.5 and 1.8, resulting in a K_liver_ value of 1.25. A study in six dairy cows and one bull (Houben et al. 2025) corroborated those ratios, finding a liver-serum ratio range of 0.9 to 3.1 after long-term exposure. In contrast, liver-plasma ratios in the single bolus cattle data (Lupton et al. 2014, 2015) used in the present study ranged between 0.23 and 1.00; thus, this discrepancy led to overprediction. Since the cattle studies were single dose, PFOS may not have distributed in a consistent manner into tissues as it would in a steady-state scenario.

The observed plasma concentration data in cattle was irregular, with rapid decreases after the absorption phase, and peaks in concentration in the distribution phase multiple weeks after dosing. Figure 3b showed a prolonged distribution phase of up to 100 days, while Fig. 3c showed an outlier peak at 42 days. Lupton et al. (2014) and Lupton et al. (2015) hypothesize that a redistribution from tissues was the main driver for those secondary peaks. The authors of the latter study also stated that control animals grazing with the test animals showed increasing levels of PFOS in plasma over time. This indicated that the animals may have reabsorbed PFOS by coming into contact with contaminated feces and urine, leading to heightened concentrations.

### Sheep

Tissue concentrations in the dairy sheep studies were underpredicted by more than a factor of four. This contrasted the beef cattle studies, which used identical K_tissue_ values, where tissue concentrations were overpredicted. The liver-plasma concentration ratios found in the fitting data (Kowalczyk et al. 2012) ranged approximately between 4 and 5, which was considerably higher than the K_liver_ value used. Due to phylogenetic proximity and similarity in tissue composition of sheep and cattle, similar concentration ratios would be expected in both species. In the validation data, the predicted liver concentrations were in the range of the observed data, but the predicted liver concentration decreased more slowly than the observed concentration. This suggests the presence of additional elimination processes in liver tissue that the model did not account for.

The fits to sheep data matched the highest plasma concentration levels; however, the total amounts of PFOS excreted were underestimated (OR Table S6). When F_a_ and k_a_ fitted values from cattle data were used, overall concentrations were lower due to the higher fecal excretion resulting from a lower F_a_ estimate in cattle (0.95) compared to sheep (0.97). In that case, the plasma concentrations during the exposure phase of the 1.16 μg/kg sheep were predicted accurately, while the concentrations of the 1.45 μg/kg sheep were underpredicted.

### Chicken

Yeung et al. (2009) remarked that the ten-fold difference in dosing between the 100 and 1000 μg/kg studies led to a ten-fold increase in liver and kidney concentrations, whereas blood concentration only increased six-fold. The source of this non-linearity is unknown. Kidney concentrations were generally predicted in the range of the observed data, while liver concentrations were overpredicted by up to a factor of 12. The subcutaneous study (Yoo et al. 2009), used as a reference for K_liver_, reported an average liver-blood concentration ratio of 9, while the oral validation study (Yeung et al. 2009) reported a maximal ratio of 1.4. This discrepancy was visible when comparing the predictions resulting from K_liver_, to the observed liver concentrations of the oral study in Fig. 8a, b. Both studies examined the same breed (White Leghorn) and used similar experimental and housing conditions. Yeung et al. (2009) hypothesized that differences in results compared to Yoo et al. (2009) were because of differences in dosing route, magnitude, and duration, and also because of differences in the age of the animals. A study on laying chicken (Kowalczyk et al. 2020) gave a liver-blood concentration ratio of 2.3 (assuming BP = 0.45), closer to the observed values of the validation data. However, that study was not considered for the calculation of K_tissue_ values, as 99% of PFOS was found in egg yolk, thus not reflecting the kinetics of non-laying chicken.

### Growth model

Simulations accounting for BW gain showed more plausible blood/plasma elimination phases and more accurate tissue concentration predictions. This was especially pronounced in the cattle validation study, with differences in FEs between the growth and static BW models of up to 30% in plasma and 80% in liver, over the span of a year. The influence of growth dilution on the liver concentrations can be clearly seen in the predictions of the sheep validation data (Fig. 6c), where the predicted concentrations of the static BW model remained at a constant level. The tissue concentration predictions in chickens between the growth and static BW models differed by factors greater than two.

The effect of growth was compared to the static BW model only during the depuration phase. Since exposure to PFOS was BW dependent, the differences between predictions would be even more pronounced if BW were also held constant during exposure. As animals eat more with increasing weight, exposure is increased, and tissue volumes and biological activity are affected. Livestock is bred to grow rapidly; thus, a growth-integrated PBK model allows for more accurate simulation of exposures lasting from infancy to maturity.

While there exist several PBK models in humans that account for growth (Gastellu et al. 2025), literature on such models in animals is scarce. A PBK model for dairy cattle (Moenning et al. 2023) reported changes in body composition of calves by linearly increasing adipose share in tissue over time. Grech et al. (2019) described a model in fish that included the effects of growth. The present model used the Richards growth curve, which has commonly been applied for modeling growth in livestock (Thornley and France 2007), lending to the interpretability of its parameters as opposed to, for example, polynomial fits. As the parameters for the Richards curve have previously been estimated (Inauen et al. 2025), parameter values optimally describing BW gain in study animals can conveniently be plugged in. Tissue volumes were taken as a fixed fraction of BW, which was a reasonable assumption in chickens, as Yeung et al. (2009) reported no significant changes in liver/kidney-to-BW ratios over 42 days. However, for cattle and sheep, this was a simplified assumption; Moallem et al. (2004) showed that in young heifers, blood mass scaled linearly with BW gain while liver, heart, kidney, and intestines masses scaled in a sublinear manner. In the present model, the tissue fractions were estimated from adult populations; to incorporate a nonlinear relationship between BW and tissue volume, the respective fractions would require recalibration.

### PFOS models in literature

Several kinetic models for PFOS have been developed in recent years, of which an overview is given in OR Table S8. Chou et al. (2023) developed a generic PBK model for PFOS, PFOA, and PFHxs exposure in adult beef and dairy cattle. EHC and fecal excretion from the intestines were modeled, as well as saturable tubular reabsorption of renally filtered PFOS. Experimental data, such as the beef cattle studies treated in the present study (Lupton et al. 2014, 2015), were used to fit several parameters, such as K_muscle_, biliary excretion, and transporter values of renal reabsorption. Their model overpredicted the liver concentration at day 105 of the high-dose long-term study (Lupton et al. 2015) in beef cattle, which was the same issue encountered in this study. A population toxicokinetic one-compartmental model for beef and dairy cattle by Mikkonen et al. (2023) modeled concentration-time curves of serum, liver, and muscle, using the Brody curve (Brody 1945) to simulate growth. Feed intake was assumed to be time-dependent on seasons. Volume of distribution was fitted, and K_tissue_ values were obtained from literature resources which overlapped with the literature data used for this study. Thus, the authors used similar K_tissue_ values as the present study, which did not lead to systematic bias in their predictions when compared to observed data.

Fischer et al. (2025) used in vitro transporter values of EHC and tubular reabsorption in mice to simulate concentrations of different PFAS in blood and tissue. In addition, binding in tissue was considered. Notably, it was found that 92% of PFOS in mice was excreted renally. Deepika et al. (2021) presented an age-dependent PBK model in humans, scaling weight, height, blood flow, and tissue volumes over time with a polynomial regression fitted to literature growth data. Growth of volumes of specific tissues was modeled using tissue-specific data, thus not assumed to be directly proportional to BW, unlike in the present model. Further, a decrease in renal filtration with increasing BW, an increase in tubular reabsorption capability with increasing BW up to maturity, and higher f_up_ in children and geriatrics was assumed. The main elimination route was modeled to be renal with saturable reabsorption and did not include EHC or fecal excretion. The authors assumed a decrease in renal filtration with increasing age due to loss of kidney nephrons in humans after the age of 30 (Noronha et al. 2022), while the present model positively correlated the renally cleared amounts with growth due to increases in kidney size. Due to the short lifespan of livestock relative to humans, the decrease in kidney function was not a factor for the present study. PBK models in rats and monkeys assumed that saturability of tubular reabsorption played an important role in the elimination of PFOS (Tan et al. 2008; Loccisano et al. 2012); the same idea was also adopted in Deepika et al. (2021) and Chou et al. (2023). Louisse et al. (2023) found that in humans, PFOS is a substrate of OAT4 for renal reabsorption with estimated in vitro values of 2.2 nmol/min/mgprotein (Jmax) and 24 mg/L (Km). The estimated human Jmax value in the present study was 20 times smaller than the one used in Chou et al. (2023), and magnitudes larger than the one used in Deepika et al. (2021).

Literature on EHC in livestock is chemical-specific and lacking regarding PFOS. In humans, PFOS was found to be a substrate of Na^+^/taurocholate cotransporting polypeptide, apical sodium-dependent bile salt transporter, and the organic solute transporter α/β subunits responsible for the EHC of bile acids (Zhao et al. 2015). In rats and humans, 95–97% of PFOS were estimated to be reabsorbed from the gut (Harada et al. 2007), consistent with the estimates of Fa in this study. Elimination routes of PFOS differ per species: in mice and rats, the main route is via urine, and to a lesser extent via feces (Cui et al. 2010; Chang et al. 2012; Fischer et al. 2025), while in humans, as well as in swine, fecal excretion takes precedence over urinary excretion (Harada et al. 2007; Numata et al. 2014). In larger species such as monkeys, cattle, and sheep (Tan et al. 2008; Kowalczyk et al. 2012; Lupton et al. 2014), renally excreted PFOS was only a minimal percentage of dose (monkeys: < 0.1% per day, cattle: 0.5% per 28 days, and not quantifiable in sheep). In milk-producing species (dairy sheep, dairy cattle) and humans, small amounts of PFOS are also transferred into milk (Kowalczyk et al. 2012, 2013; Vestergren et al. 2013; González and Domingo 2025); while in egg-producing species (laying hens), practically all PFOS is transferred into eggs (Kowalczyk et al. 2020). Lupton et al. (2014) found no metabolites of PFOS in beef cattle, indicating that metabolism is not relevant for PFOS elimination, consistent with findings in other species such as rats and primates (European Food Safety Authority (EFSA) 2008). Due to the limited data available, additional studies are required to assess excretion routes with confidence (Death et al. 2021).

### Partition coefficients

The highest concentrations of PFOS are generally found in blood and tissue such as liver, kidney, bile, and muscle; the exact distribution varies by species (Houde et al. 2006; Death et al. 2021). As a general pattern in this study, it was found that for tissue-plasma ratios, liver > kidney > muscle. For accurate predictions in tissue, the choice of K_tissue_ values is important. Ideally, in vivo K_tissue_ values are calculated from animals where PFOS is assumed to have an equilibrium concentration between plasma and tissues. Due to the availability of multiple data sources in liver, kidney, and muscle at steady state, in vivo partition coefficients were used for those tissues for cattle and sheep; for the other tissues, in silico estimates were used instead. For consistency, the same procedure was applied for chicken, where only one literature source was available (Yoo et al. 2009), which, however, led to inaccurate predictions in the liver. The high variability of tissue concentrations may stem from the differences in study design and analytical methods. To achieve more specific results, K_tissue_ values may be fitted to observed data as implemented in Chou et al. (2023), but the number of studies suited for that task is minimal.

### Limitations

Next to the choice of K_tissue_ values, prediction inaccuracies may arise due to the simplicity of the structure which does not account for all physiological processes. The lack of specificity and reliance on observed data may add uncertainty about the parameter estimates during model fitting. An example was the estimate of the absorption rate constant k_a_, which, due to the EHC of PFOS, played an important role in determining accumulation. Absorption half-life varied between cattle, sheep, and chickens, with estimates of 59, 7, and 139 h, respectively. An even higher absorption half-life of 415 h in chicken was reported by Tarazona et al. (2015) after employing a one-compartmental model. Ruminants such as cattle and sheep have a distinctively different ingestion process compared to chickens, which store feed in a crop, delaying absorption (Wood et al. 1963). Since in the generic model, k_a_ determines initial absorption through the crop, stomach, and intestines, but also reabsorption in the intestines after bile excretion, the parameter estimate of k_a_ in chicken may not reflect physiological absorption rates. More detailed and physiologically plausible modeling of EHC is possible (Okour and Brundage 2017), nevertheless, this simplified implementation of the GIT was chosen from a parsimonious and genericness standpoint. To increase confidence in estimates of k_a_, intravenous exposure data is necessary (Li and Jusko 2023).

## Conclusions

The presented approach enabled PBK modeling of growing livestock, from birth to maturity. Incorporation of growth improved the accuracy of predictions substantially, especially over extended periods, as seen, for example, in the 80% FE difference between the growth and static BW models predicting liver concentrations in cattle (Lupton et al. 2015). With the physiologically-based nature of the model, not only plasma time-concentrations but also tissue concentrations and excretion amounts were predicted. By combining growth with the EHC and tubular reabsorption structure, the fits to feces and urine data were sufficient to predict plasma concentrations in cattle, with more than 70% of predicted data within a two-fold error. For sheep and chickens, less excretion data was available, but the same principle could be applied in theory. For predictions in tissue, more data is needed to increase confidence in K_tissue_ values. The genericness of the model allows for fast translation to multiple animal species and other PFASs or environmental chemicals.

## Supplementary Information

Below is the link to the electronic supplementary material.

Supplementary Material 1

Supplementary Material 2

## Data Availability

Additional online resources are provided at 10.5281/zenodo.17251105.

## Abbreviations

A: Asymptotic weight Richards curve
AUC: Area under the curve
BP: Blood–plasma ratio
BW: Body weight
C_liver_: Liver concentration
CL_renal_: Renal clearance
CO: Cardiac output
d: Shape parameter Richards curve
EHC: Enterohepatic circulation
F_a_: Fraction absorbed
FE: Fold error
f_up_: Fraction unbound in plasma
GIT: Gastro-intestinal tract
GMFE: Geometric mean fold error
GSA: Global sensitivity analysis
Jmax: Maximal OAT4 transporter rate
k_a_: Oral absorption rate constant
Km: Michaelis constant for OAT4 transport
K_tissue_: Tissue-plasma partition coefficient
kU: Slope at the inflection point Richards curve
lbfgsb3c: Limited-memory Broyden–Fletcher–Goldfarb–Shanno bound constrained algorithm
logP: Log octanol-water partition coefficient
OAT4: Organic anion transporter 4
OR: Online resource
PBK: Physiologically based kinetic model
PFAS: Per- and polyfluoroalkyl substances
PFHxs: Perfluorohexanesulfonic acid
PFOA: Perfluorooctanoic acid
PFOS: Perfluorooctanesulfonic acid
pKa: Negative logarithmic acid dissociation constant
Q_bile_: Bile flow
Q_milk_: Milk flow
Q_tissue_: Tissue blood flow
Q_urine_: Urine flow
V_tissue_: Tissue volume
W_0_: Birth weight Richards curve
