# Supplementary material for: Implementation of growth curves in a generic physiologically based kinetic model with a case study of perfluorooctanesulfonic acid (PFOS) in beef cattle, sheep, and chicken
Source: Arch Toxicol. 2026 May 2;100(8):3443–61. doi: 10.1007/s00204-026-04421-z (PMC13379421; doi:10.1007/s00204-026-04421-z)
Supplement: Supplementary file 1 — Supplementary Material 1 [file 204_2026_4421_MOESM1_ESM.docx]

Supplementary material

# PBK model

Tissue concentration ($C_{\mathrm{tissue}}$) was calculated as PFOS amount ($A_{\mathrm{tissue}}$) divided by tissue volume ($V_{\mathrm{tissue}}$). Venous blood concentration leaving tissues ($CV_{\mathrm{tissue}}$) was calculated as tissue concentration divided by the respective plasma partition coefficient $K_{\mathrm{tissue}}$ multiplied by the blood-plasma ratio (BP):

$$CV_{\mathrm{tissue}}=C_{\mathrm{tissue}}/K_{\mathrm{tissue}}*BP$$

Venous blood concentration (CV) was defined as

$$CV=\left( \left( Q_{\mathrm{liver}}+Q_{\mathrm{gut}} \right)*CV_{\mathrm{liver}}+\sum_{\mathrm{tissue}} Q_{\mathrm{tissue}}*CV_{\mathrm{tissue}} \right)/Q_{\mathrm{cardiac}}$$

with tissue consisting of kidney, adipose tissue, bone, brain, heart, lung, muscle, and in the case of dairy sheep, mammary. Q_cardiac_ is the cardiac output. Rate of change in a compartment was given as difference between arterial blood concentration $CV_{\mathrm{art}}$ and $CV_{\mathrm{tissue}}$, multiplied by blood flow to tissue (Q_tissue_). Milk, respectively, urine concentrations were calculated as $C_{\mathrm{milk}/\mathrm{urine}}=\frac{A_{\mathrm{milk}/\mathrm{urine}}}{V_{\mathrm{milk}/\mathrm{urine}}}$, with $V_{\mathrm{milk}/\mathrm{urine}}=Q_{\mathrm{milk}/\mathrm{urine}}*timestep$, and $Q_{\mathrm{milk}/\mathrm{urine}}$ being the milk, respectively, urine production. Timestep was given as one hour.

Table S1 Mass differential equations of the generic PBK model

| Compartment | Equation |
| --- | --- |
| Gut lumen | $\frac{\mathrm{dA}_{\mathrm{lumen}}}{\mathrm{dt}}=-F_{a}*k_{a}*A_{\mathrm{lumen}}+Q_{\mathrm{bile}}*BW*\mathrm{CV}_{\mathrm{liver}}-\left( 1-F_{a} \right)*k_{a}*A_{\mathrm{lumen}}$ |
| Gut tissue | $\frac{dA_{\mathrm{gut}}}{\mathrm{dt}}=Q_{\mathrm{gut}}*\left( C_{\mathrm{art}}-CV_{\mathrm{gut}} \right)+F_{a}*k_{a}*A_{\mathrm{lumen}}$ |
| Excreted into feces | $\frac{dA_{\mathrm{fecalex}}}{\mathrm{dt}}=\left( 1-F_{a} \right)*k_{a}*A_{\mathrm{lumen}}$ |
| Liver | $\frac{\mathrm{dA}_{\mathrm{liver}}}{\mathrm{dt}}=Q_{\mathrm{liver}}*\left( C_{\mathrm{art}}-\mathrm{CV}_{\mathrm{liver}} \right)+Q_{\mathrm{gut}}*\left( \mathrm{CV}_{\mathrm{gut}}-\mathrm{CV}_{\mathrm{liver}} \right)-Q_{\mathrm{bile}}*BW*\mathrm{CV}_{\mathrm{liver}}$ |
| Adipose tissue, Bone, Brain, Heart, Lung, Muscle | $\frac{dA_{\mathrm{tissue}}}{\mathrm{dt}}=Q_{\mathrm{tissue}}*\left( C_{\mathrm{art}}-CV_{\mathrm{tissue}} \right)$ |
| Kidney | $\frac{\mathrm{dA}_{\mathrm{kidney}}}{\mathrm{dt}}=Q_{\mathrm{kidney}}*\left( C_{\mathrm{art}}-\mathrm{CV}_{\mathrm{kidney}} \right)-\mathrm{CL}_{\mathrm{renal}}*BW*\frac{\mathrm{CV}}{\mathrm{BP}}*f_{\mathrm{up}}+\frac{\mathrm{Jmax}_{\mathrm{invivo}}}{Km+C_{\mathrm{urine}}}*C_{\mathrm{urine}}*\left( \frac{\mathrm{BW}}{70} \right)^{0.75}$ |
| Urine (Reservoir compartment) | $\frac{\mathrm{dA}_{\mathrm{urine}}}{\mathrm{dt}}=\mathrm{CL}_{\mathrm{renal}}*BW*\frac{\mathrm{CV}}{\mathrm{BP}}*f_{\mathrm{up}}-\frac{\mathrm{Jma}x_{\mathrm{invivo}}}{Km+C_{\mathrm{urine}}}*C_{\mathrm{urine}}*\left( \frac{\mathrm{BW}}{70} \right)^{0.75}- C_{\mathrm{urine}}*Q_{\mathrm{urine}}$ |
| Excreted into urine | $\frac{dA_{\mathrm{urineex}}}{\mathrm{dt}}=C_{\mathrm{urine}}*Q_{\mathrm{urine}}$ |
| Blood | $\frac{\mathrm{dA}_{\mathrm{Blood}}}{\mathrm{dt}}=Q_{\mathrm{cardiac}}*\left( CV-C_{\mathrm{art}} \right)$ |
| Mammary | $\frac{\mathrm{dA}_{\mathrm{mammary}}}{\mathrm{dt}}=Q_{\mathrm{mammary}}*\left( C_{\mathrm{art}}-\mathrm{CV}_{\mathrm{mammary}} \right)-Q_{\mathrm{milk}}*C_{\mathrm{mammary}}$ |
| Milk (Reservoir compartment) | $\frac{\mathrm{dA}_{\mathrm{milk}}}{\mathrm{dt}}=Q_{\mathrm{milk}}*C_{\mathrm{mammary}}-Q_{\mathrm{milk}}* C_{\mathrm{milk}}$ |
| Excreted into milk | $\frac{\mathrm{dA}_{\mathrm{milkex}}}{\mathrm{dt}}=Q_{\mathrm{milk}}* C_{\mathrm{milk}}$ |

*Table S2 Physiological PBK model parameters for cattle, sheep and chicken*

| Type | Parameter | Unit | Beef cattle | Ref. | Dairy/Non-dairy sheep | Ref. | Chicken | Ref. |
| --- | --- | --- | --- | --- | --- | --- | --- | --- |
| Physiological | V_blood_ | %BW | 4.40 | (Dorne et al. 2023) | 5.85/5.99 | (Dorne et al. 2023) | 8.06 | (Dorne et al. 2023) |
|  | V_adipose_ | %BW | 21.25 |  | 24.05/24.58 |  | 12.14 |  |
|  | V_brain_ | %BW | 0.12 |  | 0.31/0.32 |  | 0.30 |  |
|  | V_bone_ | %BW | 14.78 |  | 12.33/12.6 |  | 23.15 |  |
|  | V_heart_ | %BW | 0.46 |  | 0.57/0.58 |  | 0.68 |  |
|  | V_gut_ | %BW | 1.96 |  | 6.80/6.95 |  | 4.54 |  |
|  | V_kidney_ | %BW | 0.28 |  | 0.31/0.32 |  | 0.95 |  |
|  | V_liver_ | %BW | 1.50 |  | 1.89/1.93 |  | 2.72 |  |
|  | V_lung_ | %BW | 0.96 |  | 1.38/1.42 |  | 0.93 |  |
|  | V_muscle_ | %BW | 54.29 |  | 44.32/45.31 |  | 46.50 |  |
|  | V_other_ | %BW | - |  | 2.19/ - |  | - |  |
|  | CO | L/h/kg | 6.82 |  | 6.60/6.60 |  | 9.95 |  |
|  | Q_adipose_ | %CO | 9.28 |  | 2.02/2.16 |  | 1.92 |  |
|  | Q_bone_ | %CO | 4.09 |  | 5.79/6.19 |  | 15.84 |  |
|  | Q_brain_ | %CO | 1.23 |  | 1.65/1.76 |  | 0.50 |  |
|  | Q_heart_ | %CO | 2.73 |  | 3.95/4.22 |  | 7.02 |  |
|  | Q_gut_ | %CO | 29.33 |  | 33.69/36.03 |  | 22.61 |  |
|  | Q_kidney_ | %CO | 9.55 |  | 12.46/13.32 |  | 14.6 |  |
|  | Q_liver_ | %CO | 4.23 |  | 2.28/2.44 |  | 8.43 |  |
|  | Q_lung_ | %CO | 3.82 |  | 2.54/2.72 |  | 3.83 |  |
|  | Q_muscle_ | %CO | 35.74 |  | 29.13/31.15 |  | 25.29 |  |
|  | Q_mammaries_ | %CO | - |  | 6.49/ - |  | - |  |
|  | Q_urine_ | L/h | 1.125 | (Edwards et al. 2015) | 0.115 | (Marsden et al. 2020) | 0.0228 | (Sommerville and Fox 1987) |
|  | Q_bile_ | L/h/kg | 0.00084 | (Symonds et al. 1982) | 0.00044 | (Heath et al. 1970) | 0.00047 | (Lisbona et al. 1981) |
|  | Q_milk_ | L/h | - | - | 0.054 | (Sinclair et al. 2010; Carcangiu et al. 2013; Castro-Costa et al. 2014; Pazzola et al. 2014; Thomas et al. 2014; Morsy et al. 2016) | - | - |

# Partition coefficients

## Cattle

For references Vestergren et al. (2013) and Lupton et al. (2025), partition coefficients K_tissue_ were calculated as tissue-to-blood/plasma concentration ratios. Since for Vestergren et al. (2013), individual data was not available, standard deviations (SD) of the ratios could not be directly calculated. To find an estimate of the SD for the ratios, 1000 correlated random samples of tissue and blood concentrations were generated assuming a log-normal distribution with the given means and SDs. Liver and blood concentrations were assumed to be correlated with a Pearson coefficient of 0.8; for kidney and blood concentrations a coefficient of 0.75 was assumed (Lupton et al. 2025). The ratio of the tissue and blood concentrations was taken, and the SDs were computed. Random effects mean of all studies and between-study variance ($\tau^{2}$) (REML estimator) were computed using the ‘metamean’ function in the R package meta (v8.0-2).

Table S3 Means and SDs of plasma K_tissue_ values from literature references in cattle

| Reference | n | K_liver_ | K_kidney_ | K_muscle_ | Original matrix | Note |
| --- | --- | --- | --- | --- | --- | --- |
| (Vestergren et al. 2013) | 5 | 0.53 (0.078*) | - | 0.081 (0.059*) | Blood | Calculated from tissue-to-blood ratio by multiplying with BP (0.45) (Ehresman et al. 2007). |
| (Lupton et al. 2025) | 5 | 1.84 (0.19) | 0.24 (0.08) | 0.04 (0.004) | Plasma | Heifers 2 weeks after depuration start |
| (Lupton et al. 2025) | 5 | 1.38 (0.19) | 0.27  (0.07) | 0.05 (0.01) | Plasma | Heifers 20-22 weeks after depuration start |
| (Drew et al. 2022) | 12 | 0.87 (0.13) | 0.4 (0.04) | 0.072 (0.018) | Serum | Heifers 63 days after depuration start. Serum-to-tissue ratio assumed to be the same as plasma-to-tissue ratio (Ehresman et al. 2007). |
| (Drew et al. 2022) | 5 | 1.64 (0.19) | 0.58 (0.13) | 0.08 (0.028) | Serum | Heifers 215 days after depuration start. Serum-to-tissue ratio assumed to be the same as plasma-to-tissue ratio (Ehresman et al. 2007). |
| Random effects mean (95% confidence intervals) | 32  (27 kidney) | 1.25 (0.77-1.73) | 0.37 (0.22-0.51) | 0.06 (0.043-0.077) |  |  |
| Between-study variance $\boldsymbol{\tau}^{\boldsymbol{2}}$ | 32  (27 kidney) | 0.29 | 0.02 | 0.0003 |  |  |

*SD estimated.

## Chicken

Plasma K_tissue_ values were calculated as tissue-to-blood concentration ratios and then multiplied by BP (0.45).

Table S4 Literature plasma partition coefficients chicken

| Reference | n | K_liver_ | K_kidney_ | K_brain_ | Original matrix | Note |
| --- | --- | --- | --- | --- | --- | --- |
| (Yoo et al. 2009) | 6 | 6.5232 | 0.28 | 0.06 | Blood | White Leghorn low dose (subcutaneous). |
| (Yoo et al. 2009) | 6 | 1.989 | 0.37 | 0.36 | Blood | White Leghorn high dose (subcutaneous). |
| Mean | 12 | 4.2561 | 0.325 | - |  | - |

# Growth


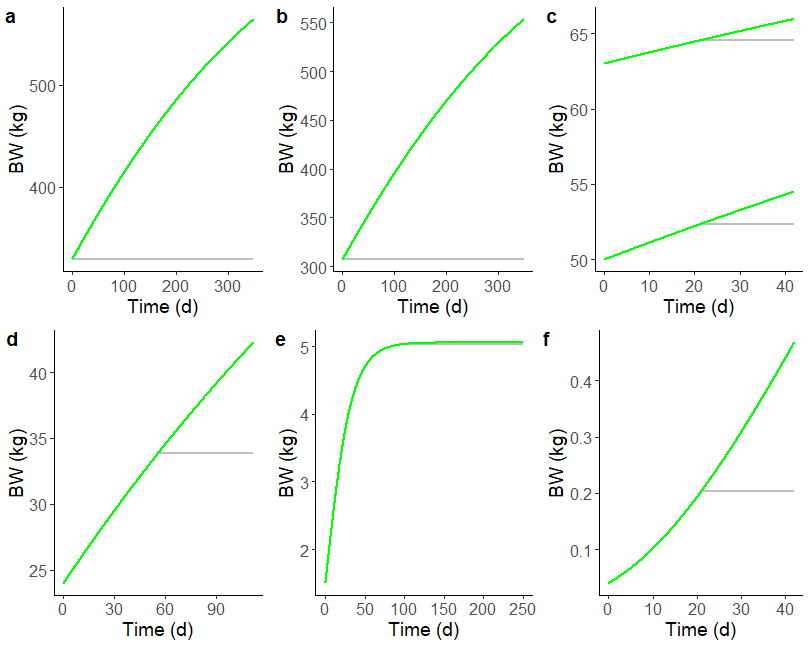


Fig. S1 Simulated BW gain. a) beef cattle(Lupton et al. 2014), b) beef cattle (Lupton et al. 2015), c) dairy sheep (Kowalczyk et al. 2012) for 1.16 μg/kg/day and 1.45 μg/kg/day dosing sheep, d) non-dairy sheep (Zafeiraki et al. 2016), e) broiler chickens (Tarazona et al. 2015) f) male laying chickens (Yeung et al. 2009). The green curves are the simulated growth curves arising from the Richards curve. The grey lines are the simulations where BW is held constant after end of exposure.

Table S5 BW gain data of the treated studies

| Species | n | Study duration  (days) | Estimated age (days) | Mean BW study begin observed (kg) | Mean BW study end observed (kg) | Mean BW study end predicted (kg) | Reference |
| --- | --- | --- | --- | --- | --- | --- | --- |
| Beef cattle | 3 | 28 | 320 | 329 | - | 354 | (Lupton et al. 2014) |
| Beef cattle | 4 | 343 | 297 | 308 | - | 550 | (Lupton et al. 2015) |
| Dairy sheep | 2 | Sheep 1:  42  Sheep 2:  21 | Sheep 1: 264  Sheep 2:  404 | Sheep 1: 50  Sheep 2: 63 | - | Sheep 1: 54.5  Sheep 2: 64.5 | (Kowalczyk et al. 2012) |
| Sheep | 8 | 112 | 91 | 24 | 40 | 42.3 | (Zafeiraki et al. 2016) |
| Broiler chicken | 6 | 231 | 25 | 1.5 | 5.15 | 5.06 | (Tarazona et al. 2015) |
| Male layer chicken | 12 | 42 | 2 | 0.04 | 0.475 | 0.467 | (Yeung et al. 2009) |

# Saturable reabsorption kinetics

Renal organic anion transporter 4 (OAT4)-mediated uptake of PFOS in humans (Louisse et al. 2023)

***In vitro* values:**

Km: 48 μM = 24 mg/L (assuming molecular weight PFOS: 500000 mg/mol)

Jmax_invitro_: 2.2 nmol/min/mgprotein

**Scaling to human *in vivo*:**

- OAT4 uptake mainly in kidney cortex (Yang et al. 2010; Prasad et al. 2016)
- Mean kidney cortex weight: 105000 mg (Kumar et al. 2018) (210000 mg for two kidney cortexes)
- Protein content in kidney cortex: 0.3 mgprotein/mgcortex (Kumar et al. 2018)

Thus, scaling to *in vivo* value:

$$Jmax_{invivo}$$

$$=Jmax_{invitro}*Proteincontent*Cortexwt*Molecularwt*timeconversion =\frac{\frac{2.2*{10}^{-9} mol}{min}}{mgprotein}*\frac{0.3 mgprotein}{mgcortex}*210000 mgcortex*500000\frac{mg}{mol}*\frac{60min}{h}= 4158\frac{mg}{h}$$

This is used as initial value for Jmax_invivo_ while fitting the model for cattle.

# Model fits to feces and urine data

Table S6 Observed and fitted accumulated excreted amounts of PFOS for beef cattle (28 days) and dairy sheep (21 days) of the growth PBK model

|  |  | **Urine (μg)** | | **Feces (μg)** | | **Milk (μg)** | |  |
| --- | --- | --- | --- | --- | --- | --- | --- | --- |
| **Species** | **n** | **Observed** | **Fitted** | **Observed** | **Fitted** | **Observed** | **Fitted** | **Reference** |
| **Beef cattle** | 3 | 14500 | 16794 | 291100 | 282009 | - | - | (Lupton et al. 2014) |
| **Dairy sheep 1** | 1 | - | 2 | 53 | 47 | 25 | 10 | (Kowalczyk et al. 2012) |
| **Dairy sheep 2** | 1 | - | 2 | 89 | 74 | 26 | 12 | (Kowalczyk et al. 2012) |
| **Dairy sheep 1 (Cattle Fa, ka values)** | 1 | - | 1 | 53 | 67 | 25 | 7 | (Kowalczyk et al. 2012) |
| **Dairy sheep 2 (Cattle Fa, ka values)** | 1 | - | 2 | 89 | 106 | 26 | 9 | (Kowalczyk et al. 2012) |

# Cattle to sheep translation of ka and Fa estimates


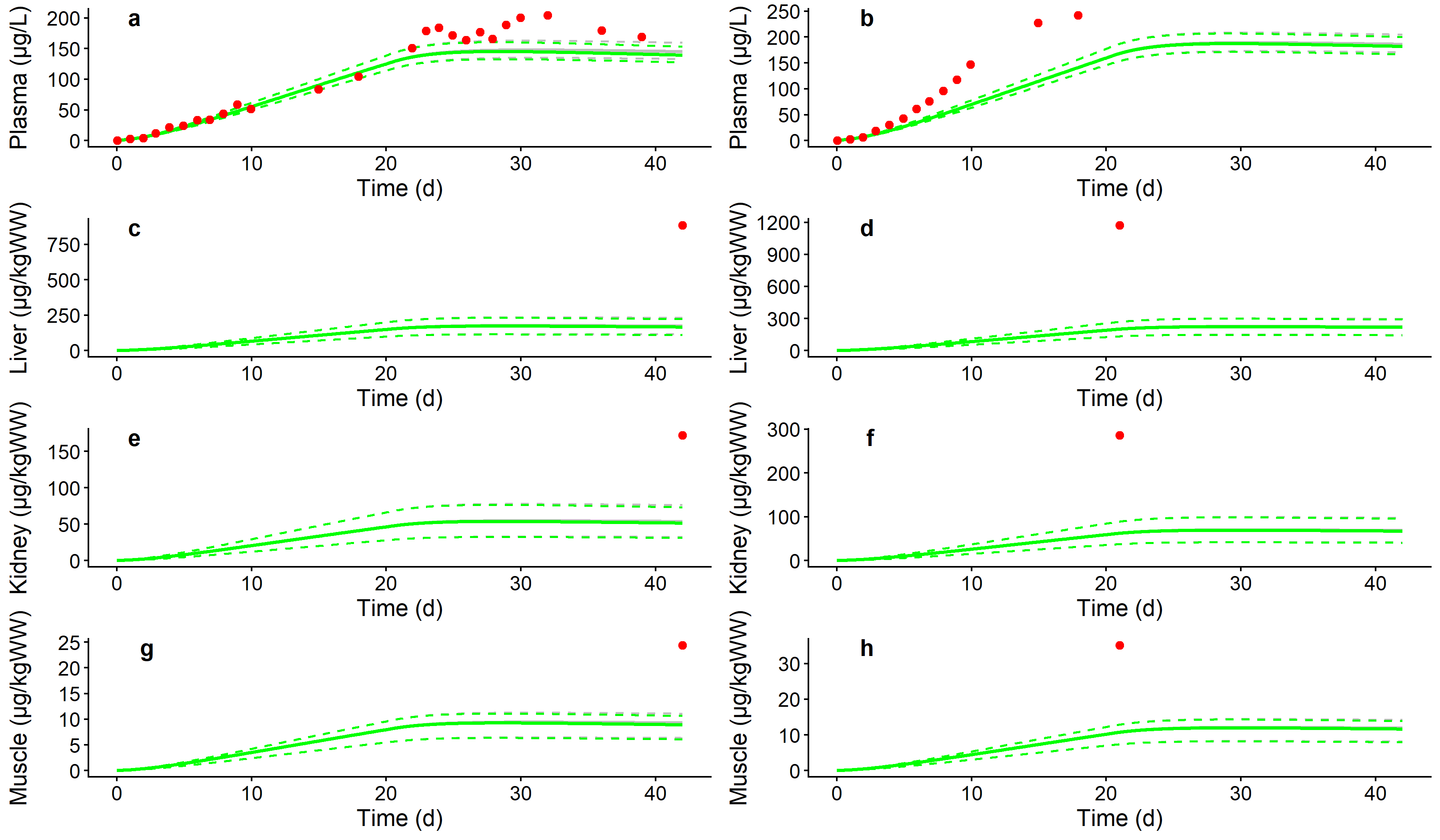


Fig. S4 Observed plasma and tissue data, and simulated time-concentration curves after multiple oral exposures, using cattle estimates for ka and Fa. Fitting data in dairy sheep 1.16 μg/kg/day: a) plasma, c) liver, e) kidney, g) muscle, and 1.45 μg/kg/day: b) plasma, d) liver, f) kidney, h) muscle (Kowalczyk et al. 2012). Tissue concentrations are given per kg wet weight (WW). The red dots are the mean observed data points. The green curves represent the median simulation results including growth and the gray curves are the results where BW was held constant after exposure. The dashed curves are the results of the introduced variation of the partition coefficients.


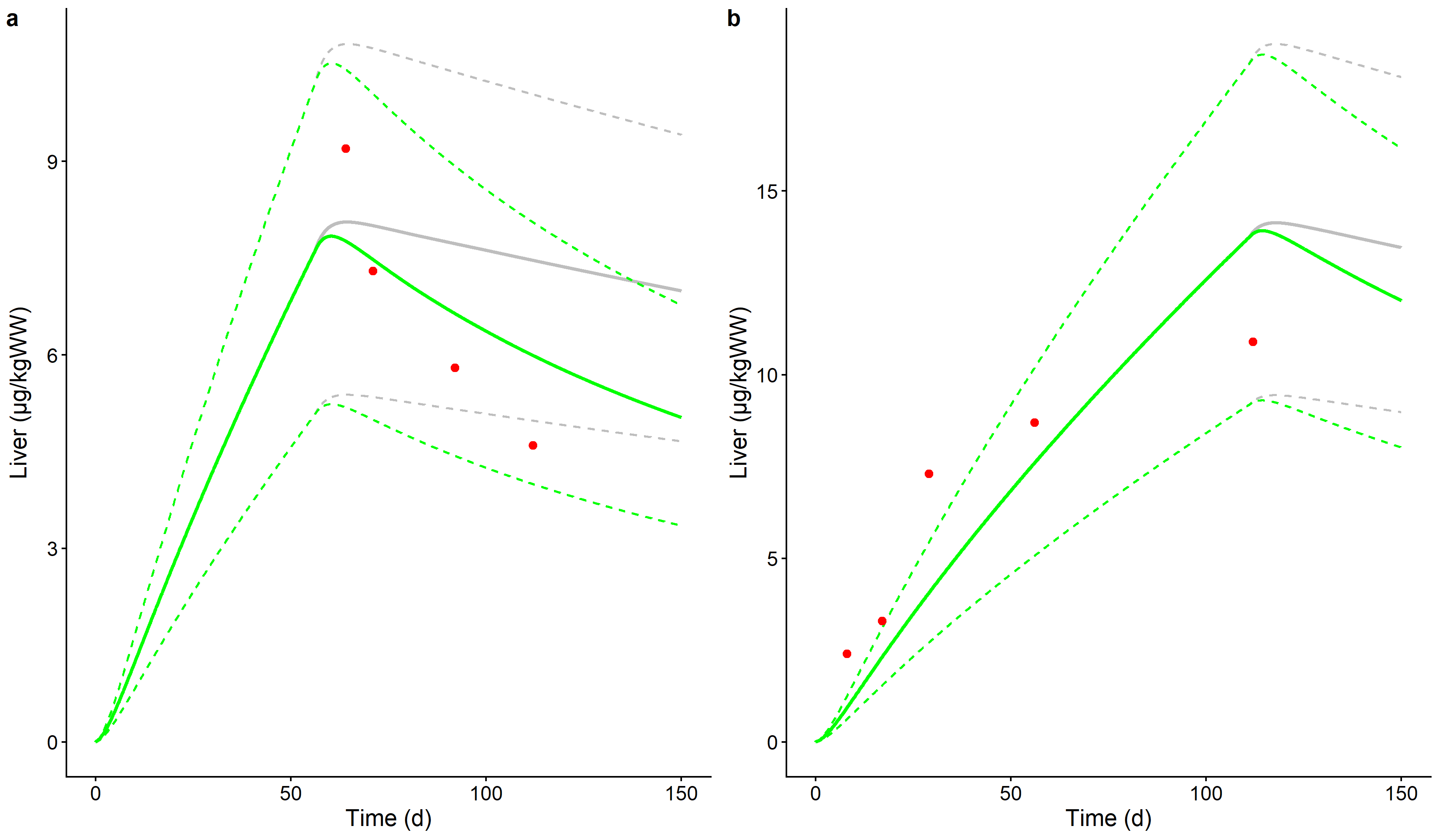


Fig. S5 Observed liver data and simulated time-concentration curves after multiple oral exposures, using cattle estimates for ka and Fa. Validation data in non-dairy sheep: 0.0208 μg/kg/day, a) 56 days exposure, b) 112 days exposure (Zafeiraki et al. 2016). Tissue concentrations are given per kg wet weight (WW). The red dots are the mean observed data points. The green curves represent the median simulation results including growth and the gray curves are the results where BW was held constant after exposure. The dashed curves are the results of the introduced variation of the partition coefficients.

*Table S7 Evaluation metrics of predicted tissue concentrations in the fitting and validation data of sheep, using cattle estimates for ka and Fa. For time-concentration curves, the GMFE is given with % two-fold in parentheses. For singular time point measurements, the time of measurement is given as well as the FE. For sheep liver time-concentration of the validation data, the time range is given*

| Reference | (Kowalczyk et al. 2012) | (Kowalczyk et al. 2012) | (Zafeiraki et al. 2016) | (Zafeiraki et al. 2016) |
| --- | --- | --- | --- | --- |
| Species | Female dairy sheep | Female dairy sheep | Sheep | Sheep |
| n animals | 1 | 1 | 4 | 4 |
| Exposure | 1.16 μg/kg/day | 1.45 μg/kg/day | 0.0208 μg/kg/day up to day 56 | 0.0208 μg/kg/day up to day 112 |
| Plasma/Blood | 1.21 (100%) | 1.63 (84.6%) | - | - |
| Plasma/Blood, BW constant | 1.20 (100%) | 1.63 (84.6%) | - | - |
| Measurement time points (days) | 42 | 21 | 56-112 | 0-112 |
| Liver | 0.19 | 0.17 | 1.16 (100%) | 1.58 (80%) |
| Liver, BW constant | 0.20 | 0.17 | 1.28 (100%) | 1.58 (80%) |
| Kidney | 0.30 | 0.22 | - | - |
| Kidney, BW constant | 0.31 | 0.22 | - | - |
| Muscle | 0.37 | 0.31 | - | - |
| Muscle, BW constant | 0.39 | 0.31 | - | - |

# Global sensitivity analysis


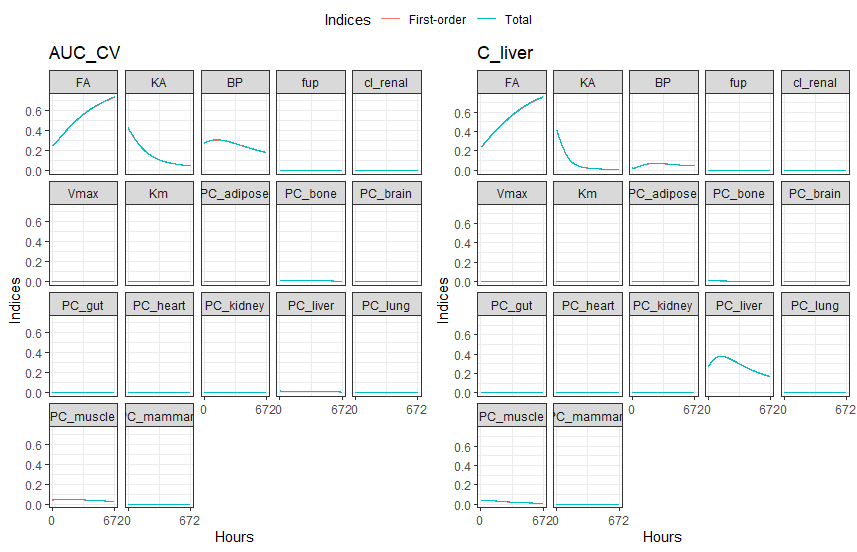


Fig. S6 Sobol first-order and total indices for beef cattle (Lupton et al. 2014) with kinetic endpoints AUC blood and liver concentration.


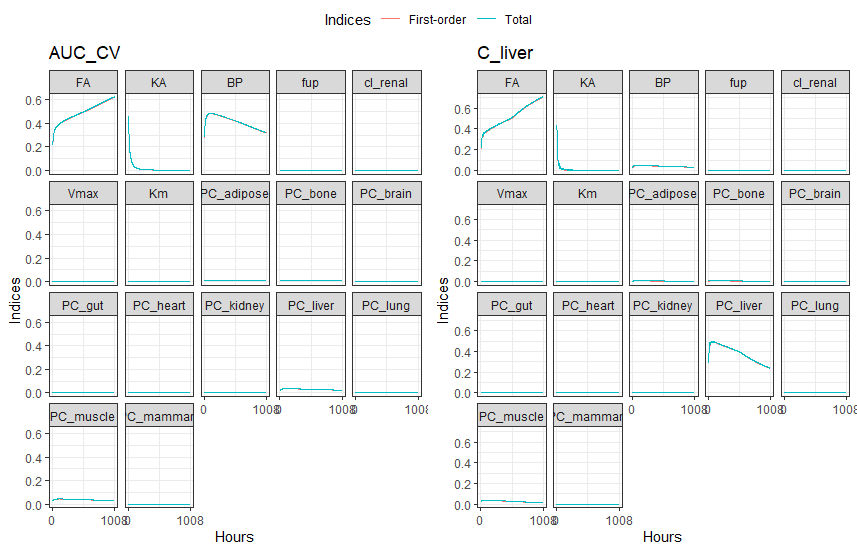


Fig. S7 Sobol first-order and total indices for dairy sheep (1.16 μg/kg) study (Kowalczyk et al. 2012) with kinetic endpoints AUC blood and liver concentration.


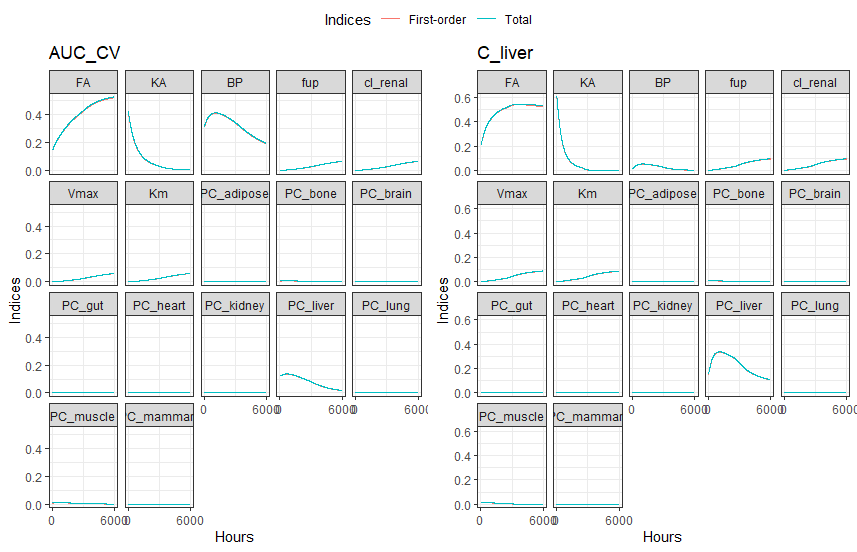


Fig. S8 Sobol first-order and total indices for broiler (Tarazona et al. 2015) with kinetic endpoints AUC blood and liver concentration

# Literature models

Table S8 Comparison of recently published kinetic PFOS models

| Model | Present study | (Chou et al. 2023) | (Mikkonen et al. 2023) | (Deepika et al. 2021) | (Fischer et al. 2025) |
| --- | --- | --- | --- | --- | --- |
| Species | Beef cattle, chicken,  (dairy) sheep | Beef cattle, dairy cattle | Beef cattle, dairy cattle | Human | Mice |
| Structure | PBK | PBK | Population one compartmental | PBK | PBK |
| Elimination routes | Feces, urine, milk (dairy sheep) | Feces, urine, milk (dairy cattle) | Generic | Urine | Feces, Urine |
| Enterohepatic recirculation | Fitted fraction absorbed | (Chou et al. 2022) | Not applicable | Not applicable | (Cao et al. 2022) |
| Human Vmax reabsorption | *In vitro* :  2.2 nmol/min/mgprotein OAT4 ((Louisse et al. 2023))  *In vivo*:  4158 mg/h (initial value) | *In vitro*:  51.8 nmol/min/mgprotein  (Chou and Lin 2019) | Not applicable | *In vivo*:  0.0086 mg/h ((Loccisano et al. 2011; Fàbrega et al. 2016)) | OAT3-mediated permeability : 0.00000113 cm/s |
| Km reabsorption | ((Louisse et al. 2023)) : 24 mg/L | 775 mg/L (fitted) | Not applicable | 0.000018 mg/L (Fàbrega et al. 2016) |  |
| Partition coefficients | (Vestergren et al. 2013; Lupton et al. 2025; Drew et al. 2022; Yoo et al. 2009), QIVIVEtools | Partially fitted (Drew et al. 2019) | (Vestergren et al. 2013; Drew et al. 2022; Lupton et al. 2015; Kowalczyk et al. 2013; Drew and Hagen 2020) | (Loccisano et al. 2013; Fàbrega et al. 2016; Rovira et al. 2019) | Multiple literature references |
| Growth | Sigmoidal (Richards) | Not applicable | Brody | Polynomial | Not applicable |
| Physico-chemical parameters scaled with growth | Urinary elimination, bile flow | Not applicable | Not applicable | Fraction unbound in plasma, urinary elimination, tubular renal resorption | Not applicable |

# References

Cao, Huiming, Zhen Zhou, Zhe Hu, et al. 2022. “Effect of Enterohepatic Circulation on the Accumulation of Per- and Polyfluoroalkyl Substances: Evidence from Experimental and Computational Studies.” *Environmental Science & Technology* 56 (5): 3214–24. https://doi.org/10.1021/acs.est.1c07176.

Carcangiu, Vincenzo, Maria Consuelo Mura, Cinzia Daga, et al. 2013. “Association between SREBP-1 Gene Expression in Mammary Gland and Milk Fat Yield in Sarda Breed Sheep.” *Meta Gene* 1 (December): 43–49. https://doi.org/10.1016/j.mgene.2013.10.001.

Castro-Costa, A., G. Caja, A. A. K. Salama, M. Rovai, C. Flores, and J. Aguiló. 2014. “Thermographic Variation of the Udder of Dairy Ewes in Early Lactation and Following an *Escherichia Coli* Endotoxin Intramammary Challenge in Late Lactation.” *Journal of Dairy Science* 97 (3): 1377–87. https://doi.org/10.3168/jds.2013-6978.

Chou, Wei-Chun, and Zhoumeng Lin. 2019. “Bayesian Evaluation of a Physiologically Based Pharmacokinetic (PBPK) Model for Perfluorooctane Sulfonate (PFOS) to Characterize the Interspecies Uncertainty between Mice, Rats, Monkeys, and Humans: Development and Performance Verification.” *Environment International* 129 (August): 408–22. https://doi.org/10.1016/j.envint.2019.03.058.

Chou, Wei-Chun, Lisa A. Tell, Ronald E. Baynes, et al. 2022. “An Interactive Generic Physiologically Based Pharmacokinetic (igPBPK) Modeling Platform to Predict Drug Withdrawal Intervals in Cattle and Swine: A Case Study on Flunixin, Florfenicol, and Penicillin G.” *Toxicological Sciences: An Official Journal of the Society of Toxicology* 188 (2): 180–97. https://doi.org/10.1093/toxsci/kfac056.

Chou, Wei-Chun, Lisa A. Tell, Ronald E. Baynes, et al. 2023. “Development and Application of an Interactive Generic Physiologically Based Pharmacokinetic (igPBPK) Model for Adult Beef Cattle and Lactating Dairy Cows to Estimate Tissue Distribution and Edible Tissue and Milk Withdrawal Intervals for per- and Polyfluoroalkyl Substances (PFAS).” *Food and Chemical Toxicology* 181 (November): 114062. https://doi.org/10.1016/j.fct.2023.114062.

Deepika, Deepika, Raju Prasad Sharma, Marta Schuhmacher, and Vikas Kumar. 2021. “Risk Assessment of Perfluorooctane Sulfonate (PFOS) Using Dynamic Age Dependent Physiologically Based Pharmacokinetic Model (PBPK) across Human Lifetime.” *Environmental Research* 199 (August): 111287. https://doi.org/10.1016/j.envres.2021.111287.

Dorne, Jean Lou C. M., José Cortiñas-Abrahantes, Fotis Spyropoulos, et al. 2023. “TKPlate 1.0: An Open-Access Platform for Toxicokinetic and Toxicodynamic Modelling of Chemicals to Implement New Approach Methodologies in Chemical Risk Assessment.” *EFSA Journal* 21 (11): e211101. https://doi.org/10.2903/j.efsa.2023.e211101.

Drew, R., and T.G. Hagen. 2020. “Factual Report for PFAS Cattle Research Project.” ToxConsult document.

Drew, R., T.G. Hagen, D. Champness, and A. Sellier. 2019. “PFAS Tissue Distribution in Cattle.” Poster Presentation at IUTOX 15th International Congress of Toxicology.

Drew, Roger, Tarah G. Hagen, David Champness, and Amelie Sellier. 2022. “Half-Lives of Several Polyfluoroalkyl Substances (PFAS) in Cattle Serum and Tissues.” *Food Additives & Contaminants. Part A, Chemistry, Analysis, Control, Exposure & Risk Assessment* 39 (2): 320–40. https://doi.org/10.1080/19440049.2021.1991004.

Edwards, GR, RH Bryant, N Smith, et al. 2015. “Milk Production and Urination Behaviour of Dairy Cows Grazing Diverse and Simple Pastures.” *Proceedings of the New Zealand Society of Animal Production* 75.

Ehresman, David J., John W. Froehlich, Geary W. Olsen, Shu-Ching Chang, and John L. Butenhoff. 2007. “Comparison of Human Whole Blood, Plasma, and Serum Matrices for the Determination of Perfluorooctanesulfonate (PFOS), Perfluorooctanoate (PFOA), and Other Fluorochemicals.” *Environmental Research* 103 (2): 176–84. https://doi.org/10.1016/j.envres.2006.06.008.

Fàbrega, Francesc, Martí Nadal, Marta Schuhmacher, José L. Domingo, and Vikas Kumar. 2016. “Influence of the Uncertainty in the Validation of PBPK Models: A Case-Study for PFOS and PFOA.” *Regulatory Toxicology and Pharmacology* 77 (June): 230–39. https://doi.org/10.1016/j.yrtph.2016.03.009.

Fischer, Fabian C., Colin Thackray, Nicholas Ferguson, et al. 2025. “Understanding Mechanisms of PFAS Absorption, Distribution, and Elimination Using a Physiologically Based Toxicokinetic Model.” *Environmental Science & Technology* 59 (26): 13240–50. https://doi.org/10.1021/acs.est.5c05473.

Heath, T., I. W. Caple, and P. M. Redding. 1970. “Effect of the Enterohepatic Circulation of Bile Salts on the Flow of Bile and Its Content of Bile Salts and Lipids in Sheep.” *Quarterly Journal of Experimental Physiology and Cognate Medical Sciences* 55 (2): 93–103. https://doi.org/10.1113/expphysiol.1970.sp002067.

Kowalczyk, Janine, Susan Ehlers, Peter Fürst, Helmut Schafft, and Monika Lahrssen-Wiederholt. 2012. “Transfer of Perfluorooctanoic Acid (PFOA) and Perfluorooctane Sulfonate (PFOS) From Contaminated Feed Into Milk and Meat of Sheep: Pilot Study.” *Archives of Environmental Contamination and Toxicology* 63 (2): 288–98. https://doi.org/10.1007/s00244-012-9759-2.

Kowalczyk, Janine, Susan Ehlers, Anja Oberhausen, et al. 2013. “Absorption, Distribution, and Milk Secretion of the Perfluoroalkyl Acids PFBS, PFHxS, PFOS, and PFOA by Dairy Cows Fed Naturally Contaminated Feed.” *Journal of Agricultural and Food Chemistry* 61 (12): 2903–12. https://doi.org/10.1021/jf304680j.

Kumar, Vineet, Jia Yin, Sarah Billington, et al. 2018. “The Importance of Incorporating OCT2 Plasma Membrane Expression and Membrane Potential in IVIVE of Metformin Renal Secretory Clearance.” *Drug Metabolism and Disposition* 46 (10): 1441–45. https://doi.org/10.1124/dmd.118.082313.

Lisbona, F, R Jimenez, A Esteller, and M. A Lopez. 1981. “Basal Biliary Secretion in Conscious Chicken and Role of Enterohepatic Circulation.” *Comparative Biochemistry and Physiology Part A: Physiology* 69 (2): 341–44. https://doi.org/10.1016/0300-9629(81)90305-4.

Loccisano, Anne E., Jerry L. Campbell, Melvin E. Andersen, and Harvey J. Clewell. 2011. “Evaluation and Prediction of Pharmacokinetics of PFOA and PFOS in the Monkey and Human Using a PBPK Model.” *Regulatory Toxicology and Pharmacology* 59 (1): 157–75. https://doi.org/10.1016/j.yrtph.2010.12.004.

Loccisano, Anne E., Matthew P. Longnecker, Jerry L. Campbell Jr, Melvin E. Andersen, and Harvey J. Clewell III. 2013. “Development of Pbpk Models for Pfoa and Pfos for Human Pregnancy and Lactation Life Stages.” *Journal of Toxicology and Environmental Health, Part A* 76 (1): 25–57. https://doi.org/10.1080/15287394.2012.722523.

Louisse, Jochem, Luca Dellafiora, Jeroen J. M. W. van den Heuvel, et al. 2023. “Perfluoroalkyl Substances (PFASs) Are Substrates of the Renal Human Organic Anion Transporter 4 (OAT4).” *Archives of Toxicology* 97 (3): 685–96. https://doi.org/10.1007/s00204-022-03428-6.

Lupton, Sara J., Kerry L. Dearfield, John J. Johnston, Sarah Wagner, and Janice K. Huwe. 2015. “Perfluorooctane Sulfonate Plasma Half-Life Determination and Long-Term Tissue Distribution in Beef Cattle (Bos Taurus).” *Journal of Agricultural and Food Chemistry* 63 (51): 10988–94. https://doi.org/10.1021/acs.jafc.5b04565.

Lupton, Sara J., Janice K. Huwe, David J. Smith, Kerry L. Dearfield, and John J. Johnston. 2014. “Distribution and Excretion of Perfluorooctane Sulfonate (PFOS) in Beef Cattle (Bos Taurus).” *Journal of Agricultural and Food Chemistry* 62 (5): 1167–73. https://doi.org/10.1021/jf404355b.

Lupton, Sara J., David J. Smith, Erin B. Howey, et al. 2025. “Tissue Histology and Depuration of Per- and Polyfluoroalkyl Substances (PFAS) from Dairy Cattle with Lifetime Exposures to PFAS-Contaminated Drinking Water and Feed.” *Food Additives & Contaminants: Part A* 42 (2): 223–39. https://doi.org/10.1080/19440049.2024.2444560.

Marsden, Karina A., Lucy Lush, Jon. A. Holmberg, et al. 2020. “Sheep Urination Frequency, Volume, N Excretion and Chemical Composition: Implications for Subsequent Agricultural N Losses.” *Agriculture, Ecosystems & Environment* 302 (October): 107073. https://doi.org/10.1016/j.agee.2020.107073.

Mikkonen, Antti T., Jennifer Martin, Richard N. Upton, et al. 2023. “Dynamic Exposure and Body Burden Models for Per- and Polyfluoroalkyl Substances (PFAS) Enable Management of Food Safety Risks in Cattle.” *Environment International* 180 (October): 108218. https://doi.org/10.1016/j.envint.2023.108218.

Morsy, Amr S., Yosra A. Soltan, Sobhy M. A. Sallam, Severino M. Alencar, and Adibe L. Abdalla. 2016. “Impact of Brazilian Red Propolis Extract on Blood Metabolites, Milk Production, and Lamb Performance of Santa Inês Ewes.” *Tropical Animal Health and Production* 48 (5): 1043–50. https://doi.org/10.1007/s11250-016-1054-1.

Pazzola, M., M. L. Dettori, C. Cipolat-Gotet, A. Cecchinato, G. Bittante, and G. M. Vacca. 2014. “Phenotypic Factors Affecting Coagulation Properties of Milk from Sarda Ewes.” *Journal of Dairy Science* 97 (11): 7247–57. https://doi.org/10.3168/jds.2014-8138.

Prasad, Bhagwat, Katherine Johnson, Sarah Billington, et al. 2016. “Abundance of Drug Transporters in the Human Kidney Cortex as Quantified by Quantitative Targeted Proteomics.” *Drug Metabolism and Disposition* 44 (12): 1920–24. https://doi.org/10.1124/dmd.116.072066.

Rovira, Joaquim, Maria Ángeles Martínez, Raju Prasad Sharma, et al. 2019. “Prenatal Exposure to PFOS and PFOA in a Pregnant Women Cohort of Catalonia, Spain.” *Environmental Research* 175 (August): 384–92. https://doi.org/10.1016/j.envres.2019.05.040.

Sinclair, Liam A., Weerasinghe M. P. B. Weerasinghe, Robert G. Wilkinson, Michael J. de Veth, and Dale E. Bauman. 2010. “A Supplement Containing Trans-10, Cis-12 Conjugated Linoleic Acid Reduces Milk Fat Yield but Does Not Alter Organ Weight or Body Fat Deposition in Lactating Ewes.” *The Journal of Nutrition* 140 (11): 1949–55. https://doi.org/10.3945/jn.110.126490.

Sommerville, Barbara A., and J. Fox. 1987. “Changes in Renal Function of the Chicken Associated with Calcitonin and Parathyroid Hormone.” *General and Comparative Endocrinology* 66 (3): 381–86. https://doi.org/10.1016/0016-6480(87)90248-6.

Symonds, H. W., D. L. Mather, and E. D. Hall. 1982. “Surgical Procedure for Modifying the Duodenum in Cattle to Measure Bile Flow and the Diurnal Variation in Biliary Manganese, Iron, Copper and Zinc Excretion.” *Research in Veterinary Science* 32 (1): 6–11.

Tarazona, J. V., C. Rodríguez, E. Alonso, et al. 2015. “Toxicokinetics of Perfluorooctane Sulfonate in Birds under Environmentally Realistic Exposure Conditions and Development of a Kinetic Predictive Model.” *Toxicology Letters* 232 (2): 363–68. https://doi.org/10.1016/j.toxlet.2014.11.022.

Thomas, David L., Yves M. Berger, Brett C. McKusick, and Claire M. Mikolayunas. 2014. “Dairy Sheep Production Research at the University of Wisconsin-Madison, USA – a Review.” *Journal of Animal Science and Biotechnology* 5 (1): 22. https://doi.org/10.1186/2049-1891-5-22.

Vestergren, Robin, Francis Orata, Urs Berger, and Ian T. Cousins. 2013. “Bioaccumulation of Perfluoroalkyl Acids in Dairy Cows in a Naturally Contaminated Environment.” *Environmental Science and Pollution Research* 20 (11): 7959–69. https://doi.org/10.1007/s11356-013-1722-x.

Yang, Ching-Hui, Kyle P. Glover, and Xing Han. 2010. “Characterization of Cellular Uptake of Perfluorooctanoate via Organic Anion-Transporting Polypeptide 1A2, Organic Anion Transporter 4, and Urate Transporter 1 for Their Potential Roles in Mediating Human Renal Reabsorption of Perfluorocarboxylates.” *Toxicological Sciences* 117 (2): 294–302. https://doi.org/10.1093/toxsci/kfq219.

Yeung, Leo W. Y., Eva I. H. Loi, Vicky Y. Y. Wong, et al. 2009. “Biochemical Responses and Accumulation Properties of Long-Chain Perfluorinated Compounds (PFOS/PFDA/PFOA) in Juvenile Chickens (Gallus Gallus).” *Archives of Environmental Contamination and Toxicology* 57 (2): 377–86. https://doi.org/10.1007/s00244-008-9278-3.

Yoo, Hoon, Keerthi S. Guruge, Noriko Yamanaka, et al. 2009. “Depuration Kinetics and Tissue Disposition of PFOA and PFOS in White Leghorn Chickens (*Gallus Gallus*) Administered by Subcutaneous Implantation.” *Ecotoxicology and Environmental Safety* 72 (1): 26–36. https://doi.org/10.1016/j.ecoenv.2007.09.007.

Zafeiraki, Effrosyni, Irene Vassiliadou, Danae Costopoulou, et al. 2016. “Perfluoroalkylated Substances in Edible Livers of Farm Animals, Including Depuration Behaviour in Young Sheep Fed with Contaminated Grass.” *Chemosphere* 156 (August): 280–85. https://doi.org/10.1016/j.chemosphere.2016.05.003.
